# Active learning for extracting surgomic features in robot-assisted minimally invasive esophagectomy: a prospective annotation study

**DOI:** 10.1007/s00464-023-10447-6

**Published:** 2023-10-14

**Authors:** Johanna M. Brandenburg, Alexander C. Jenke, Antonia Stern, Marie T. J. Daum, André Schulze, Rayan Younis, Philipp Petrynowski, Tornike Davitashvili, Vincent Vanat, Nithya Bhasker, Sophia Schneider, Lars Mündermann, Annika Reinke, Fiona R. Kolbinger, Vanessa Jörns, Fleur Fritz-Kebede, Martin Dugas, Lena Maier-Hein, Rosa Klotz, Marius Distler, Jürgen Weitz, Beat P. Müller-Stich, Stefanie Speidel, Sebastian Bodenstedt, Martin Wagner

**Affiliations:** 1 Department of General, Visceral and Transplantation Surgery, Heidelberg University Hospital, Heidelberg, Germany; 2 National Center for Tumor Diseases (NCT), Heidelberg, Germany; 3 Department of Translational Surgical Oncology, National Center for Tumor Diseases (NCT/UCC), Dresden, Germany; 4 German Cancer Research Center (DKFZ), Heidelberg, Germany; 5 Faculty of Medicine and University Hospital Carl Gustav Carus, Technische Universität Dresden, Dresden, Germany; 6 Helmholtz-Zentrum Dresden - Rossendorf (HZDR), Dresden, Germany; 7 Corporate Research and Technology, Karl Storz SE & Co KG, Tuttlingen, Germany; 8 Department of Intelligent Medical Systems (IMSY), German Cancer Research Center (DKFZ), Heidelberg, Germany; 9 Department of Visceral-, Thoracic and Vascular Surgery, University Hospital Carl Gustav Carus, Technische Universität Dresden, Fetscherstraße 74, 01307 Dresden, Germany; 10 Else Kröner-Fresenius Center for Digital Health, Technische Universität Dresden, Dresden, Germany; 11 National Center for Tumor Diseases (NCT/UCC), Dresden, Germany; 12 Institute of Medical Informatics, Heidelberg University Hospital, Heidelberg, Germany; 13 The Study Center of the German Surgical Society (SDGC), Heidelberg University Hospital, Heidelberg, Germany; 14 Centre for Tactile Internet With Human-in-the-Loop (CeTI), Technische Universität Dresden, 01062 Dresden, Germany; 15 University Center for Gastrointestinal and Liver Diseases, St. Clara Hospital and University Hospital Basel, Basel, Switzerland

**Keywords:** Artificial intelligence, Minimally invasive surgery, Precision medicine, Machine learning, Surgical data science, Surgomics

## Abstract

**Background:**

With Surgomics, we aim for personalized prediction of the patient's surgical outcome using machine-learning (ML) on multimodal intraoperative data to extract surgomic features as surgical process characteristics. As high-quality annotations by medical experts are crucial, but still a bottleneck, we prospectively investigate active learning (AL) to reduce annotation effort and present automatic recognition of surgomic features.

**Methods:**

To establish a process for development of surgomic features, ten video-based features related to bleeding, as highly relevant intraoperative complication, were chosen. They comprise the amount of blood and smoke in the surgical field, six instruments, and two anatomic structures. Annotation of selected frames from robot-assisted minimally invasive esophagectomies was performed by at least three independent medical experts. To test whether AL reduces annotation effort, we performed a prospective annotation study comparing AL with equidistant sampling (EQS) for frame selection. Multiple Bayesian ResNet18 architectures were trained on a multicentric dataset, consisting of 22 videos from two centers.

**Results:**

In total, 14,004 frames were tag annotated. A mean F1-score of 0.75 ± 0.16 was achieved for all features. The highest F1-score was achieved for the instruments (mean 0.80 ± 0.17). This result is also reflected in the inter-rater-agreement (1-rater-kappa > 0.82). Compared to EQS, AL showed better recognition results for the instruments with a significant difference in the McNemar test comparing correctness of predictions. Moreover, in contrast to EQS, AL selected more frames of the four less common instruments (1512 vs. 607 frames) and achieved higher F1-scores for common instruments while requiring less training frames.

**Conclusion:**

We presented ten surgomic features relevant for bleeding events in esophageal surgery automatically extracted from surgical video using ML. AL showed the potential to reduce annotation effort while keeping ML performance high for selected features. The source code and the trained models are published open source.

**Graphical abstract:**

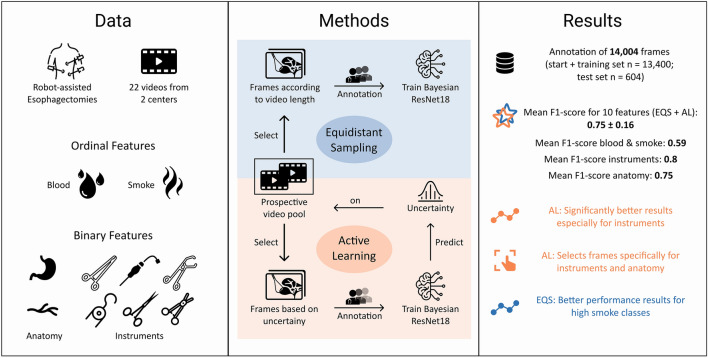

**Supplementary Information:**

The online version contains supplementary material available at 10.1007/s00464-023-10447-6.

Operating rooms are high stake environments that still lack comprehensive and real-time monitoring and evaluation [1] with intraoperative adverse events being associated with higher postoperative morbidity and mortality [2]. In the intraoperative adverse event classification by Francis et al. [3], different types of bleeding are important examples of the five grades of intraoperative adverse events, including the highest grades 4 and 5. Furthermore, the occurrence of bleeding was identified as the most frequent type of adverse events by the SEVERE score developed by Jung et al. [4]. However, currently these events of a surgical procedure must be evaluated manually by experts, which does not allow for a standardized, objective, and scalable analysis of surgical videos.

Surgical Data Science aims to address this kind of problems using machine-learning (ML) methods as a sub-discipline of artificial intelligence (AI) to extract knowledge from data [5]. Certainly, ML has shown tremendous success, also in the field of surgery [6] by applying methods like computer vision, e.g., for automatic instrument recognition [7] or surgical phase detection [8] to surgical video data. However, relevance for guiding treatment decisions for example in surgical oncology remains limited up until now [9].

The concept of Surgomics uses methods of Surgical Data Science [5] that focus especially on the intraoperative setting with the aim to enable a personalized prediction of the surgical patient's outcome [10]. Surgomic features are characteristics of a surgical procedure which are automatically derived from multimodal intraoperative data to quantify processes in the operating room. Based on the conceptual work of Wagner et al. [10], we now aimed to develop surgomic features that are automatically derived from surgical videos by means of ML. The overarching aim of our work is thus to improve surgical therapy by extracting quantitative information from surgical data that may help to predict postoperative complications. Given the relevance of intraoperative events, we decided to address an automatic analysis of occurrence and surgical management of bleeding events in the surgical field. However, training ML algorithms for automatic analysis of surgical procedures still requires high quantity and quality expert labeled data, and this remains a major bottleneck [11]. Methods like active learning (AL) [12] can be used to reduce the total annotation effort but have so far only been investigated retrospectively, but not prospectively in surgery [13]. Furthermore, to our knowledge, in this field AL has not yet been systematically compared to conventional approaches for frame selection such as equidistant sampling (EQS). While AL selects frames in a variable interval using machine intelligence, EQS selects frames within a fixed interval, for example one frame every second or every one or two minutes from a surgical video.

With the aim of automatically extracting ten surgomic features and investigating the potential of AL in this process, we addressed three major research questions in this study:

1. How well does ML automatically extract the selected ten surgomic features from frames of robot-assisted minimally invasive esophagectomies (RAMIE) videos?
2. Does AL reduce the annotation effort and show better results in comparison to EQS for frame selection in a prospective setting?
3. How can the resulting surgomic features be visualized after surgery for a comprehensive quantitative description?

## Materials and methods

### Surgomic feature selection

**Fig. 1:**
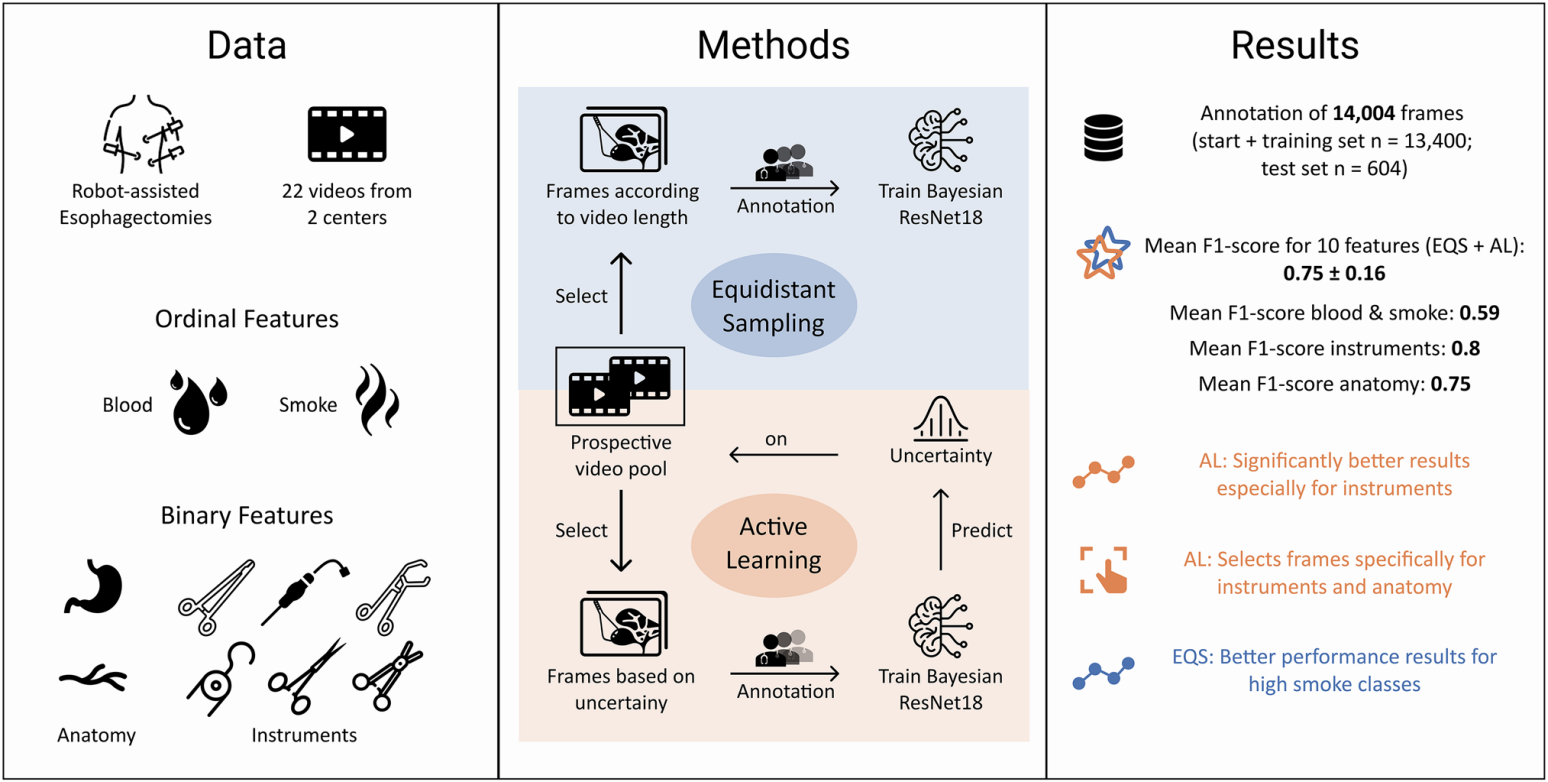
Visual abstract of the annotation study. The development process of the surgomic features with required data and selected features, experimental setup with feature annotation investigating equidistant sampling (EQS) vs. active learning (AL) for frame selection, and results are depicted

**Fig. 2:**
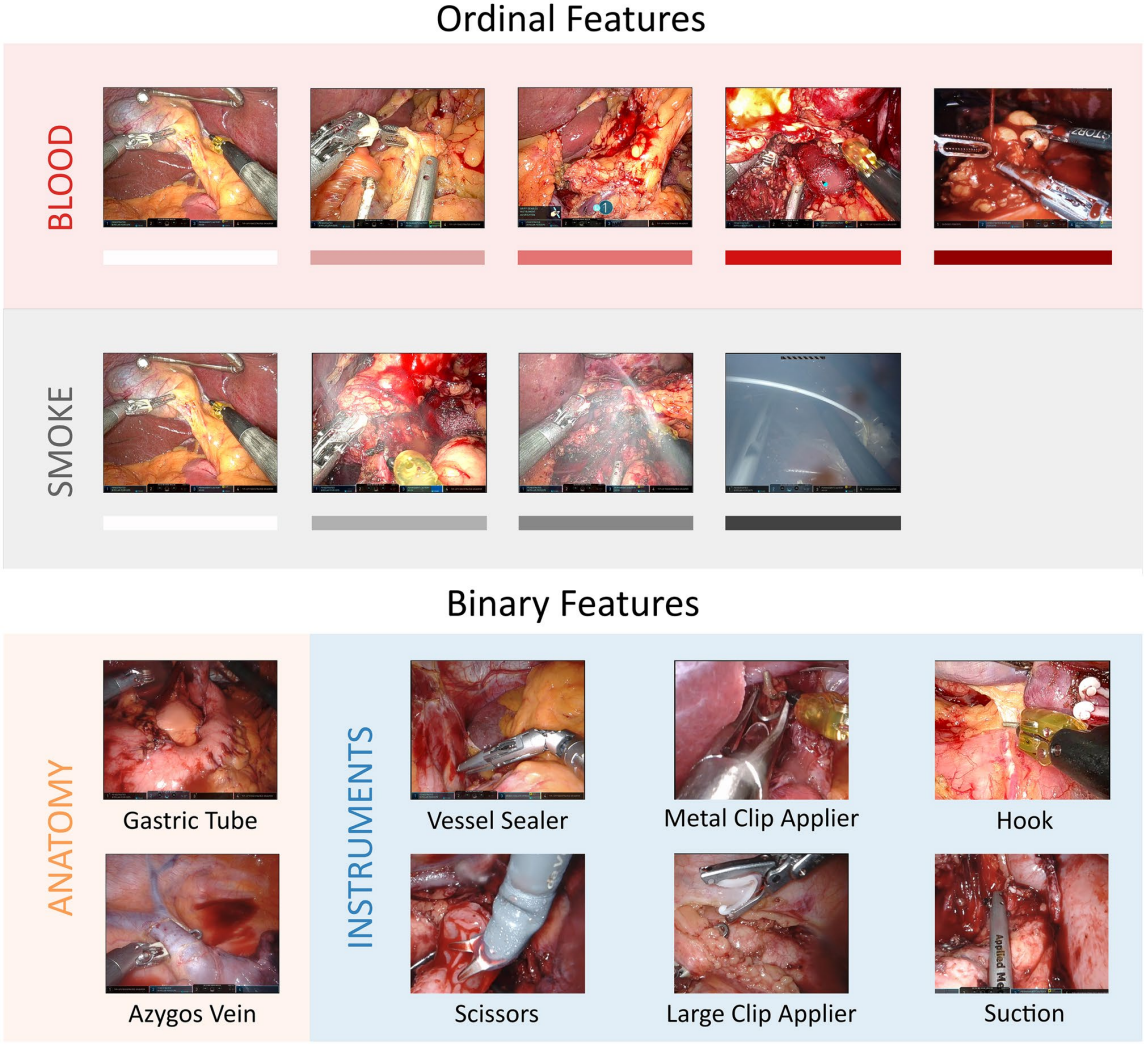
Annotation of surgomic features. Example frames for all ordinal and binary surgomic features. For the ordinal features, blood and smoke, example frames for every scale level are presented. Supplement 1 includes a detailed annotation protocol for all features

In this study we chose to focus on the extraction of surgomic features concerning intraoperative bleeding, which may result either from surgical error and/or from challenging patient characteristics. Based on this focus, we selected ten surgomic features for this study (Figs. 1 and 2) that are of particular importance for the recognition and surgical handling of bleeding. According to the work of Wagner et al. [10], the ten surgomic features were selected from two different feature categories, namely “surgical field” and “instrument”. These two categories showed the highest technical feasibility based on the judgment of (computer) scientists in previous investigations [10] and can mainly be derived from surgical videos. From the “surgical field'' category four features were selected: “blood” and “smoke” in the surgical field as well as the presence of the anatomic structures “gastric tube” and “azygos vein”. Regarding the surgomic feature blood, the Forrest classification has already been introduced for gastrointestinal hemorrhages distinguishing between active bleeding, recent bleeding, and no bleeding [14]. In analogy to this classification, we developed a scale applicable to all locations and kinds of bleeding occurring during surgery. Our blood scale reaches from zero “no blood” to four “blood amount requiring immediate intervention” (Fig. 2, supplement 1) allowing for a differentiated gradation. Regarding visibility in the surgical field, smoke is together with blood an important factor to analyze. In the present article we also developed a scale for the surgomic feature “smoke” allowing a distinction of the different amounts of smoke potentially impairing the surgery. The four-level scale ranges from zero “no smoke” to three “smoke amount leading to no visibility” (Fig. 2, supplement 1).

The two scales were developed together with surgical experts and computer scientists. The final decision on the scales was made by a board-certified surgeon. The aim was to find the best possible balance between clinically important levels (blood levels 3 and 4 need expeditious surgical intervention, smoke level 3 indicates to pause the procedure) and an assumed feasible problem solution for the neural networks. Thresholds were defined based on surgical expertise (e.g., immediate bleeding control by clip becomes necessary for blood level 4) as well as morphological image criteria (e.g., a spurting hemorrhage at blood level 4 or blurred organ margins at smoke level 2) (see supplement 1 for more details).

With RAMIE videos as a data source, the two anatomic structures gastric tube and azygos vein were chosen as surgomic features because of their visibility and importance in this intervention, as well as the risk they bear as sites of relevant bleeding. From the “instrument” category, the presence of the instruments “vessel sealer”, “permanent cautery hook”, “suction”, “scissors'', “large clip applier”, and “metal clip applier” were selected as six surgomic features. All these instruments are used during RAMIE, are relevant for the appearance of blood and smoke in the surgical field and often interact directly with the two chosen anatomic structures. For all selected frames the ten surgomic features were annotated by tag-annotation using a self-hosted version of CVAT [15], an open-source annotation tool. An annotation protocol with the ten features and their different scale levels was created for the study (supplement 1). The protocol contains a description and example frames for each feature and feature scale level.

### Active learning for efficient annotation

To automatically extract the ten surgomic features from surgical videos, we used ML. However, modern ML algorithms usually require large amounts of training data annotated by (medical) experts. With the aim to achieve similar or better ML performance with less annotations, we here investigated AL as a method for intelligent annotation. Comparative approaches have been successfully used for instrument presence and surgical phase detection [13] within surgery, however up until now only retrospectively.

We set up a prospective AL framework for the annotation of surgomic features (Fig. 1). This means, the trained ML model iteratively chose new frames to be annotated based on the hypothesis that intelligent selection of frames would result in improved performance of the ML algorithms with less annotated training data.

From a technical perspective, following the approach of [13], an architecture capable of calculating a confidence for its predictions was trained on previously labeled data (see “Training” section). Subsequently, the model’s confidence was calculated on all available unlabeled data points. After handing the most uncertain samples to the annotators and extending the labeled pool, the cycle was repeated (Fig. 1).

### Study design of prospective comparative study

To establish a dynamic feature development process and to investigate the potential role and applicability of AL in it, a prospective comparative study was designed. Here, we compared AL for intelligent selection of frames to be annotated to state-of-the-art EQS of frames from the video footage (Fig. 1). Over ten prospective annotation and training cycles, the comparison of the two methods enabled investigating whether AL improves annotation efficiency, i.e., resulting in similar or better algorithm performance with fewer annotations.

The following paragraph describes selection of ML algorithm, the surgical data set that we used, the creation of a start dataset for initial training, the ten annotation and training cycles of our comparative study and the creation of the test dataset to measure algorithm performance.

### ML algorithm selection

In this work, we opted for a ResNet architecture [16] pretrained on ImageNet, as this state-of-the-art architecture also was used by almost all participants for the instrument classification task in the 2019 EndoVis Surgical Skill and Workflow Challenge [17]. Due to the limited size of our dataset, arising from the nature of the investigated problem of annotation effort, we used the smaller ResNet18 instead of a ResNet50.

Following the approach of [18] suggesting using a smaller proxy model, improving speed, and reducing computational cost while keeping the expected performance of AL, we performed the AL cycle with a smaller image resolution of 180 by 240 pixels and a larger one of 480 by 640 for evaluation. For the larger evaluation models additional augmentation in form of random scaling, rotation, and brightness and color shift was applied to the frames. All models were trained for 100 epochs using an SGD optimizer [19] with a OneCycle learn rate scheduler [20]. The maximum learn rate was 3e–3, the batch size of 16 was determined by available GPU size. To obtain the model’s prediction certainty, which is required for AL, the model was transformed into a Bayesian model by adding Monte–Carlo dropouts as proposed in [21]. The uncertainty was calculated using the standard deviation over multiple inferences over the same sample, as described in [13]. We split the features according to their feature group, resulting in 3 models: one BayesianResNet classifying the levels of the features blood and smoke, one detecting the presence of the features of anatomic structure presence, and one detecting the presence of the features of instrument presence. As we were comparing EQS to AL, in total 6 models were trained per annotation cycle.

The code and trained models are publicly available at https://gitlab.com/nct_tso_public/active-learning-for-surgomic-features.

### Data set

As surgical data, we used 26 RAMIE videos from two different centers (13 from the Department of General, Visceral and Transplantation Surgery at the Heidelberg University Hospital and 13 from the Department of Visceral, Thoracic and Vascular Surgery at the University Hospital Carl Gustav Carus Dresden). Ethics approval was granted by the ethics committees at Heidelberg University (S-248/2021) and at the Technical University Dresden (BO-EK-177032021). For prospectively collected data, all patients provided written informed consent into use of their data. Here, 22 videos were used for training, 4 videos for testing, with each center providing half of the videos. The recorded surgical videos were either collected prospectively (n = 13) or were taken retrospectively from a prospectively collected database (n = 13). In any case, the chronological order was kept during the experiments to replicate a prospective study design. Video sequences with the laparoscopic camera filming outside the patient body were manually annotated and then before frame selection automatically replaced with completely white frames. This way, the total duration of the video remained unchanged. Respective white frames were not taken into account in the annotation process.

Two data sets were created which will be referred to in the following: a start data set used as an equal starting point for algorithm training (both AL and EQS approaches) and a test data set for performance evaluation.

The start data set was created because AL needs a pretrained model in the beginning. We created the initial start data set by equidistantly selecting frames every two minutes from the first videos of each of the two centers. This start set from those two videos resulted in 343 frames, 145 from Dresden and 198 from Heidelberg. As equidistant sampling was not able to represent rare features, one missing frame of the highest blood level had to be filled in by manual selection, was confirmed by three independent annotators, and was added to the start set for blood and smoke (n = 344). Every feature and feature level were then represented in the start set.

An independent test data set was created to evaluate the performance of the trained ML algorithms for final evaluation. In total, 604 frames for this data set were selected from additional four videos of the two centers (two from Heidelberg, two from Dresden). Of the 604 frames, 588 frames were selected equidistantly, 16 frames were selected manually to have at least three examples of each surgomic feature and each feature level in the test dataset.

### Training

Training was performed on frames of 22 videos (11 from each center). The comparative study included ten subsequent annotation cycles each for EQS and AL, simulating ML algorithms that learn in the clinic when new procedures are performed, and new videos are added. For EQS the ResNets were trained using frames selected every two minutes from the newly added video for every cycle. Frames were selected every two minutes to result with an amount of frames that can still be annotated in reasonable time due to the long duration and the high number of RAMIE videos. This way it was possible to include more videos which potentially showed variation in the procedure. For AL, frame selection was based on the uncertainty of the networks and frames were chosen from the whole available video pool growing by one video every cycle. The number of frames selected with AL was determined by how many frames were sampled equidistantly in this cycle to ensure the same amount of training data for EQS and AL. Reflecting the prospective nature of the study, nothing was changed in the setup during training, and the performance of the networks was not evaluated between cycles.

### Annotation

The group of annotators consisted of six medical experts (five medical students and one surgical resident) specifically trained with the annotation protocol. To guarantee coherence and quality of annotation for the selected frames, every training frame (including the frames of the start data set) was annotated independently by three different annotators from the group. If the three annotators did not agree, the annotation was determined using majority vote. For the ordinal features, if no majority vote was possible due to all three raters voting for different levels, the frame was discussed in a group of at least three annotators from the annotator group until agreement. During each annotation cycle a chronological order of the frames was maintained to ease especially the annotation of gastric tube and azygos vein.

The 604 frames of the test set were annotated by all six independent annotators of the annotation group to enable a more detailed evaluation of the inter-rater-reliability.

### Validation

After the ten cycles were finished, the performance was evaluated in a post hoc manner. A model with larger frames resolution was trained on the different growing data sets available after each cycle of EQS and AL and evaluated on the test set. The uncertainty was determined over 100 inferences per sample and averaged over the samples of the test set.

The test set was built of 4 videos which were held back. During the cycles the models never saw the test set. Evaluation on the test set was done by a separate script than training and frame selection.

Finally, all available annotated data combined from EQS and AL was used to train the best possible models, resulting in an upper baseline. For evaluation, the F1-score, precision and recall for every feature was calculated. The different levels of blood and smoke were aggregated hierarchically, by firstly averaging over the images and secondly averaging over the levels, resulting in the macro F1-score for those two features.

### Experimental setup

All experiments were done using Python 3.8, the models were trained and evaluated using PyTorch [22] v1.12. The metrics F1-score, precision and recall were calculated using scikit-learn [23]. Model training and inference during the AL cycles was performed on an Nvidia GTX1080 (NVIDIA Corporation, Santa Clara, California, USA).

The final model using all available data was trained on an Nvidia RTX A5000 NVIDIA Corporation, (Santa Clara, California, USA).

### Statistical analysis

To check for statistically significant differences between the trained classifiers using the AL and EQS method a McNemar test with Edwards correction [24] was performed for every feature and cycle on the test set comparing the correctness of the classifiers. Differences with a *p*-value below 0.05 are seen as significant. Additionally, the test was performed over all cycles for the feature categories: blood and smoke, instruments, and anatomy, as well as all features together, by combining the contingency tables of the respective features. The inter-rater-agreement was calculated on the test set (annotated by all six annotators) using the Kappa-Fleiss-score [25]. To evaluate different aspects three combinations were evaluated: To check for outliers within the rater group, a 1-rater-score was calculated with the kappa-score of the raw annotations of all six raters. To evaluate how many raters are needed to achieve consistent annotations, a 3-rater-score and a 5-rater-score were calculated with the kappa-score of all possible combinations to merge three/five raters. Additionally, the kappa-scores were calculated on the training sets comparing the datasets of AL and EQS after all ten cycles were finished, to check for differences in rater agreement indicating different levels of difficulty.

### Surgomic report

Finally, we created a surgomic report comprehensively presenting all features. The report was generated using matplotlib [26] in python 3.7.9 and set up in a pipeline to be automatically generated on new videos. The surgomic report contains a videogram to give a temporal overview of the video and a barcode-like plot for each feature showing the predicted presence/level for each feature over time. The videogram was generated by concatenating the middle columns of one frame per minute of the video. The surgomic features were compressed to display a total of 800 values per barcode, thus the number of predictions for one value and the duration compressed into one value differ depending on the video length. One value was calculated with the mean of the available feature predictions. The barcode was then visualized with a color-gradient representing the feature frequency. Thus, a darker line in the barcode represents more positive predictions in an interval, while a lighter color represents fewer positive predictions. Additionally, the mean certainty of the predictions for each interval is overlayed over each feature barcode. The information for each feature summarizing the whole duration of the recorded procedure can be seen in a separate box on the right. Here, the total duration of detected instruments and anatomy features in the video is displayed. For blood and smoke the total duration of high levels (levels > 2 for blood, > 1 for smoke) is shown. In addition, the mean, maximum and minimum certainty are calculated for each feature. The surgomic report with the videogram and feature barcodes can be generated after a live detection of the surgomic features in the operating room, allowing surgeons and surgical data scientists to get a direct overview of the procedure. However, the prediction certainty can only be calculated after post-processing the video offline.

### Live evaluation of surgomic features in the operating room

To test the surgomic feature prediction live in the operating room a mobile “surgomic feature tower” consisting of a medical PC, a DataLogger for recording videos, and a touch screen was installed. The used medical PC is a PANA.ceia4 (MCD Medical Computers Deutschland GmbH, Mönchengladbach, Germany, article number: 2000074 M) equipped with a Quadro RTX4000 from NVIDIA Corporation (Santa Clara, California, USA), 16 GB RAM and an Intel Core i7-8700 K processor (Intel Corporation, Santa Clara, California, USA). The DataLogger (KARL STORZ SE & Co. KG, Tuttlingen, Germany) serves as a technical platform for recording endoscopic videos of the surgery [17]. The 24.5ʺ touch screen (KARL STORZ SE & Co. KG, Tuttlingen, Germany, article number: 200905 24) allows the user to manually start and stop the feature prediction. During feature prediction, it displays features live in parallel with the endoscopic video. For the feature prediction, a set of ResNet50s without Monte–Carlo dropouts was trained on all annotated data to allow for faster inference while keeping comparable performance. The detected features such as blood and smoke are represented via a colored scale, and remaining features are highlighted via a schematic representation next to the original video. After stopping the feature prediction, the surgomic report containing the consolidated feature predictions is generated automatically.

## Results

### Frame selection

**Fig. 3:**
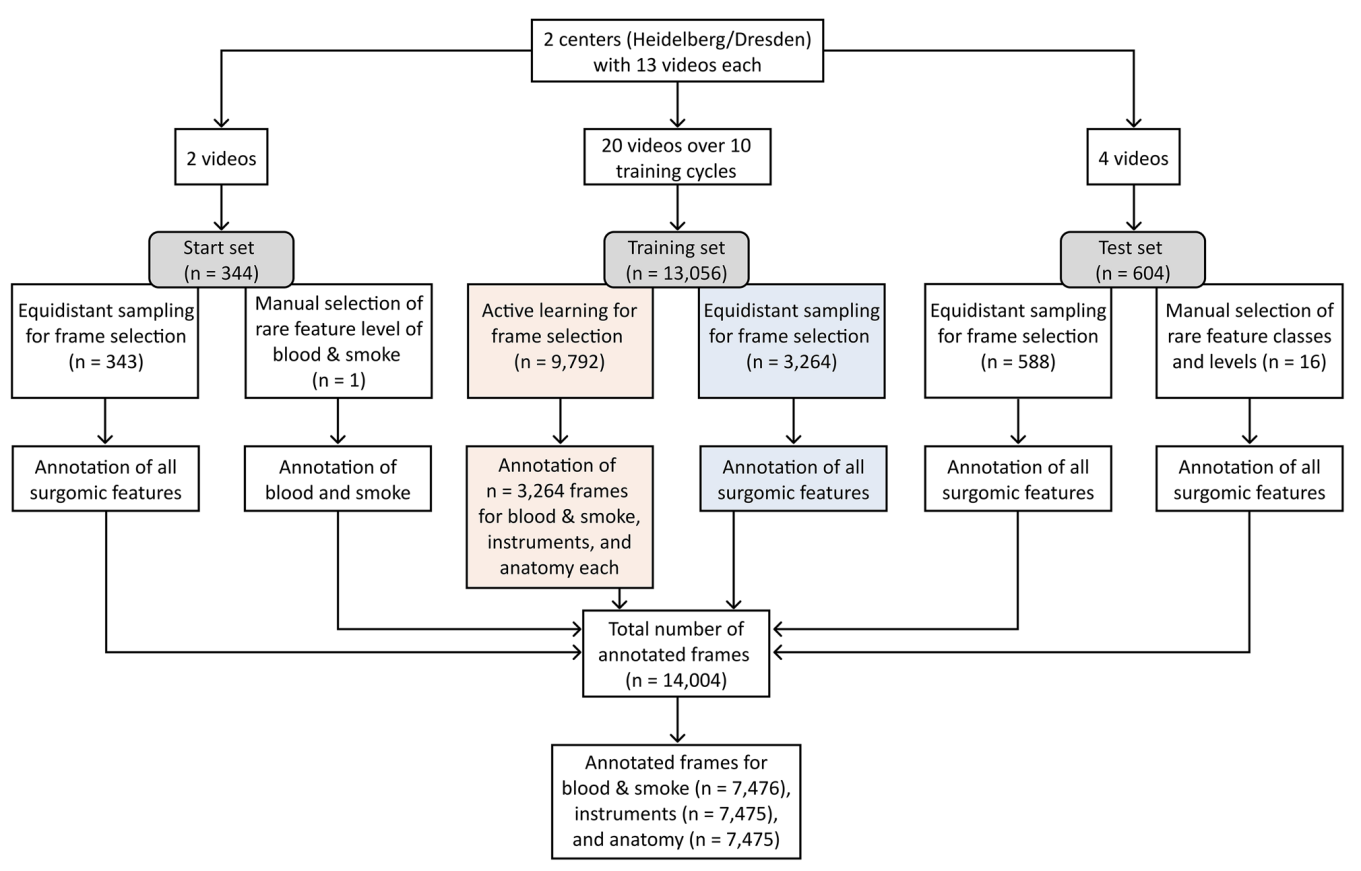
Flow diagram of the frame selection and annotation process

Over the ten cycles of EQS and AL for frame selection, a total number of 14,004 frames were annotated by a minimum of three independent raters each. Specifically, 343 frames with all features and one additional only for blood and smoke were annotated for the shared start set (Fig. 3). 604 frames were annotated for all features for the test set. For EQS 3264 frames were annotated with all features. For AL, 9792 frames (equals 3 times 3264) were annotated but not each frame with all features, because the AL algorithm was allowed to select different frames for blood/smoke, anatomy, and instruments. Overall, in the end there were three sets of frames for each feature group annotated: blood and smoke (*n* = 7476), anatomy (*n* = 7475) and instruments (*n* = 7475). Each consists of the start set, the sampled frames by EQS, the test set and for each group the respective sampled frames by AL. Figure 3 gives an overview of the number of selected and annotated frames. The affiliation of the frames to the two centers here was the following for AL: blood and smoke 2419 frames from Heidelberg, 845 from Dresden; instruments 2530 from Heidelberg, 734 from Dresden, anatomy 1995 from Heidelberg, 1269 from Dresden. For the start set and EQS, 1993 were selected from Heidelberg videos and 1615 from Dresden.

### Inter-rater-agreement

The inter-rater-agreement on the different features was evaluated using the kappa-score on the test data set that was annotated by six different annotators for each frame.

Instrument features had the highest inter-rater-agreement with 1-rater-kappa-scores of vessel sealer, permanent cautery hook and metal clip applier above 0.98. Slightly lower one-rater kappa-scores had the large clip applier, scissors, and suction, still above 0.82. A slightly worse range showed the anatomy features with one-rater kappa-scores above 0.77 for gastric tube and azygos vein.

Lowest inter-rater-agreement was observed on blood and smoke features with kappa-scores indicating that at least a 3-rater-majority or even better a 5-rater-majority vote is necessary to achieve an acceptable inter-rater-agreement (1-rater-score of 0.33 to 0.71 for blood and 0.46 to 0.65 for smoke, 3-rater-score of 0.65 to 0.84 for blood and 0.69 to 0.87 for smoke, 5-rater-score of 0.81 to 0.92 for blood and 0.87 to 0.93 for smoke). Overall, raters agreed more on binary features (instrument and anatomy presence) with a mean 1-rater score of 0.91 than on ordinal features (blood and smoke) with a mean 1-rater score of 0.52.

To further evaluate the rater agreement, the amount of complete agreement versus the need for a majority decision was evaluated on the training data set. For both anatomy features all three raters agreed in 90% of all annotated frames for EQS and 84% for AL, indicating that the annotation difficulty for the anatomic structures was higher with AL. This hypothesis was also subjectively confirmed by the raters themselves, who had mentioned even before evaluation that AL selected frames were more difficult to annotate. However, for blood and smoke as well as for instruments no difference between AL and EQS regarding the amount of complete agreement vs. majority decision could be found.

### Feature performance

**Fig. 4:**
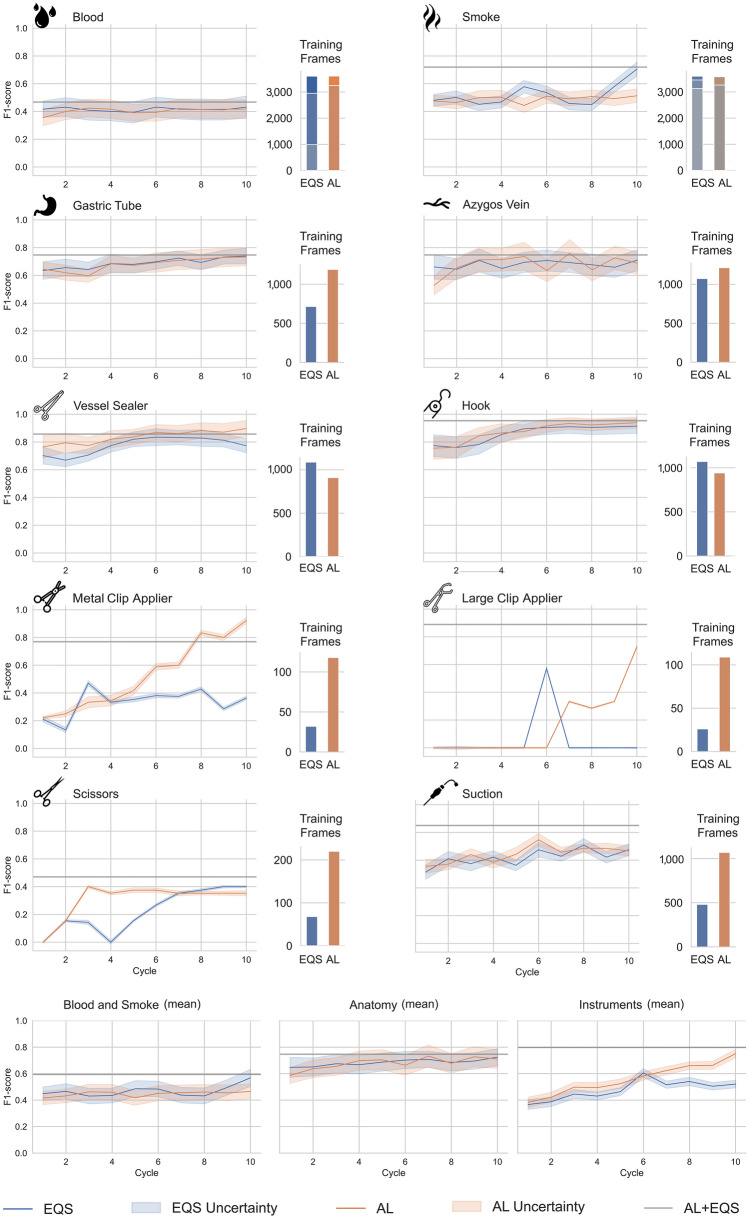
F1-scores of surgomic features for the ten cycles (line-plots) and total number of available training frames (bar plots). Equidistant sampling (EQS) for frame selection is depicted in blue and active learning (AL) in orange, the performance of the model trained on all available frames after ten cycles is shown as a reference line (AL + EQS). An error bar visualizes the uncertainty of the model, as given by the std between predictions in Bayesian models, during inference. These error bars should not be confused with confidence intervals

When training with all available annotated data from EQS and AL, recognition results were achieved with an overall hierarchically aggregated F1-score of 0.75 ± 0.16 for all 10 surgomic features (Table 1). The highest recognition results were achieved for the feature permanent cautery hook with an F1-score of 0.95, the lowest for the feature blood with an F1-score of 0.47. Among the feature groups (blood and smoke, anatomy, instruments), the instruments achieved the highest F1-score of 0.80 ± 0.17. Furthermore, the algorithms for instrument recognition showed a higher certainty in their predictions compared to anatomy, as well as blood and smoke (Fig. 4). Overall, all features achieved better results when training with both, annotated frames selected with EQS plus AL, except of the features vessel sealer and metal clip applier. For those two features AL alone achieved better results without the annotated frames of EQS. A detailed overview of the algorithms’ performances for surgomic features is shown in Table 1.

**Table 1:** Algorithm performance for surgomic features

*Cycle 10*	F1-score			Precision			Recall			Support
	EQS	AL	EQS + AL	EQS	AL	EQS + AL	EQS	AL	EQS + AL	
Blood-0	0.51	0.46	**0.57**	0.63	0.58	**0.71**	0.43	0.38	**0.48**	61
Blood-1	0.41	0.42	**0.44**	0.58	0.54	**0.63**	0.32	**0.35**	0.34	176
Blood-2	0.74	0.73	**0.74**	0.63	0.63	**0.64**	0.88	0.88	**0.89**	296
Blood-3	0.50	0.48	**0.59**	0.55	**0.63**	0.62	0.46	0.38	**0.56**	68
Blood-4	0.00	0.00	0.00	0.00	0.00	0.00	0.00	0.00	0.00	3
Blood (macro)	0.43	0.42	**0.47**	0.48	0.48	**0.52**	0.42	0.40	**0.45**	–
Smoke-0	0.97	**0.97**	0.97	**0.99**	0.99	0.99	0.95	**0.96**	0.96	552
Smoke-1	0.57	**0.64**	0.60	0.47	**0.56**	0.53	0.70	**0.73**	0.70	37
Smoke-2	**0.45**	0.44	0.38	**0.38**	0.33	0.33	0.56	**0.67**	0.44	9
Smoke-3	0.83	0.00	**0.92**	0.83	0.00	0.86	0.83	0.00	**1.00**	6
Smoke (macro)	0.70	0.51	**0.72**	0.67	0.47	**0.68**	0.76	0.59	**0.78**	–
Vessel sealer	0.77	**0.90**	0.86	0.90	0.93	**0.95**	0.68	**0.87**	0.78	161
Permanent cautery hook	0.92	0.94	**0.95**	0.90	**0.97**	**0.97**	**0.93**	0.91	**0.93**	196
Metal clip applier	0.36	**0.92**	0.77	0.40	**0.86**	0.71	0.33	**1.00**	0.83	6
Large clip applier	0.00	0.73	**0.89**	0.00	0.67	**1.00**	0.00	**0.80**	**0.80**	5
Scissors	0.40	0.35	**0.47**	**1.00**	0.60	0.80	0.25	0.25	**0.33**	12
Suction	0.68	0.67	**0.85**	0.73	0.88	**0.92**	0.63	0.53	**0.79**	43
Azygos vein	0.71	0.69	**0.75**	**0.92**	**0.92**	0.89	0.58	0.55	**0.64**	207
Gastric tube	0.74	0.73	**0.75**	0.84	**0.90**	0.89	**0.66**	0.62	0.65	144
10 Features (macro)	0.57 ± 0.27	0.69 ± 0.21	**0.75 ± 0.16**	0.68 ± 0.31	0.77 ± 0.19	**0.83 ± 0.15**	0.52 ± 0.27	0.65 ± 0.24	**0.70 ± 0.18**	–
Blood + Smoke (macro)	0.57	0.47	**0.59**	0.58	0.48	**0.60**	0.59	0.50	**0.62**	–
Instruments (macro)	0.52	0.75	**0.80**	0.66	0.82	**0.89**	0.47	0.73	**0.74**	–
Anatomy (macro)	0.72	0.71	**0.75**	0.88	**0.91**	0.89	0.62	0.59	**0.65**	–

The scores enable a comparison between AL vs. EQS for frame selection (columns EQS, AL). Additionally, networks were trained with all annotated frames combined (column EQS + AL). For every feature the F1-score as well as precision and recall are depicted for each method after ten training cycles. The best value per row and metric is bold, the second best is underlined. The number of occurrences in the test data set (support) is shown in the last column

### Equidistant sampling vs. active learning

An overall superiority of AL regarding classification performance could be shown with a mean total F1-score for all ten features after ten cycles of 0.69 ± 0.21 for AL versus 0.57 ± 0.27 for EQS (Table 1). However, this overall superiority was mainly because of the superior performance for the instrument features. Here, AL showed better results with a mean F1-score after ten cycles of 0.75 ± 0.22 (AL) vs. 0.52 ± 0.33 (EQS), precision of 0.82 (AL) vs. 0.66 (EQS) and recall of 0.73 (AL) vs. 0.47 (EQS) (Table 1). The large clip applier is to be highlighted in this regard with an F1-score of 0.73 (AL) vs. 0 (EQS) as well as the metal clip applier with an F1-score of 0.92 (AL) vs. 0.36 (EQS). However, for blood and smoke as well as for the two anatomic structures, the results for AL in comparison to EQS were similar or slightly worse. Particularly smoke was worse detected using AL with an F1-score of 0.51 (AL) vs. 0.70 (EQS). The highest smoke level had the greatest impact with an F1-score of 0.0 (AL) vs. 0.83 (EQS). For the features blood and smoke AL was not able to select specific frames of the rare feature levels like blood level 3 and 4, or smoke level 3. Here, EQS had in the end more training frames for these rare levels. The number of selected frames available for training of AL vs. EQS is shown in Fig. 4. In contrast to EQS, AL selected more frames of the four less common instruments (suction, metal clip applier, large clip applier, scissors) and the two anatomic structures. For the metal clip applier AL continuously improved performance over the cycles while EQS stagnated after cycle 4. For scissors AL stagnated after 3 cycles, but EQS took 7 cycles to reach the same performance. For the large clip applier AL was able to select samples after 7 cycles and improve the F1-score while EQS field to learn the feature.

**Fig. 5:**
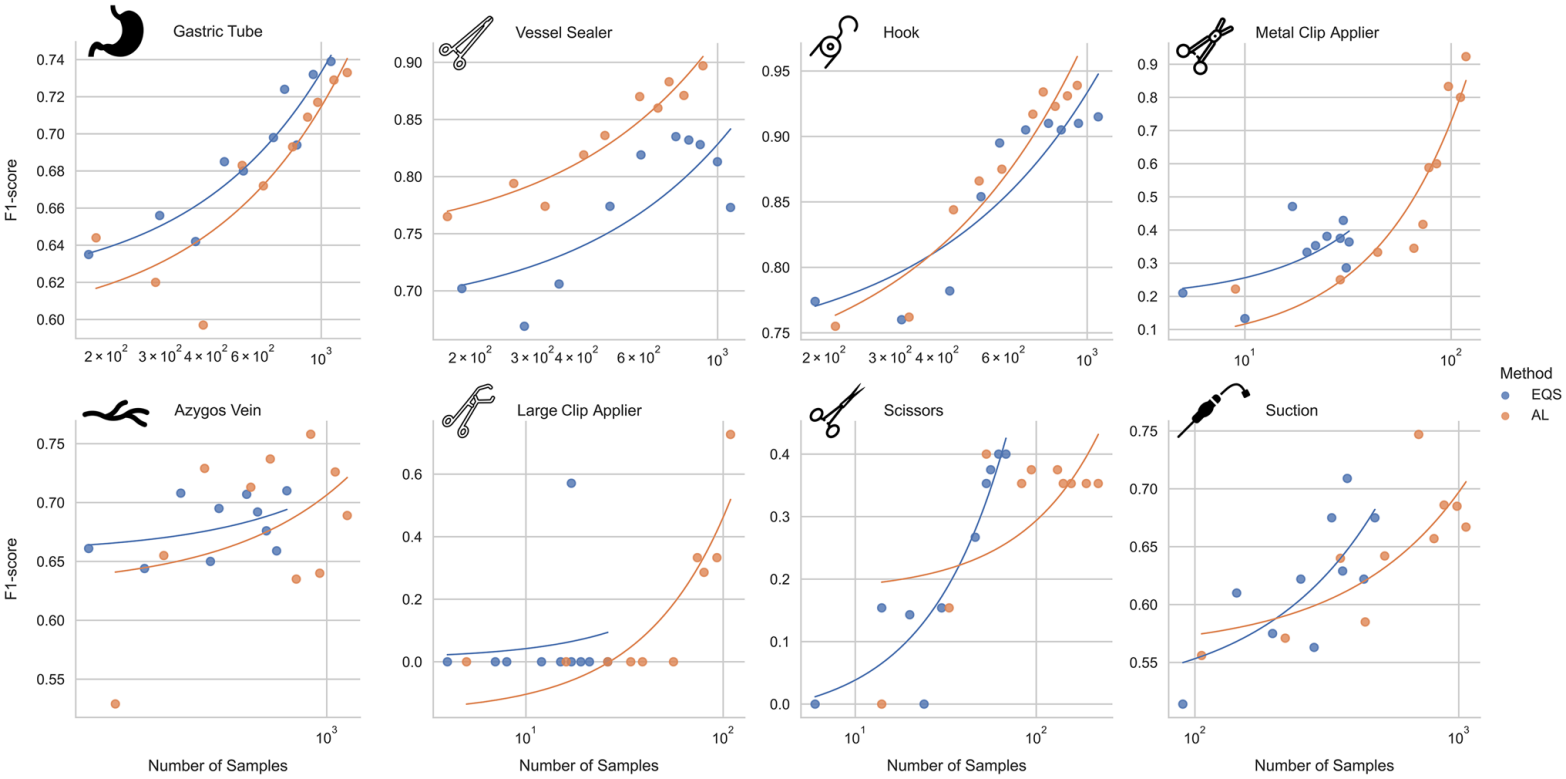
F1-scores of anatomy and instrument features in relation to the number of available positive samples. The single samples of F1-score and number of samples are plotted for all ten cycles with equidistant sampling (EQS) in blue and active learning (AL) in orange. A regression line of order 1 is shown with the same colors (Color figure online)

When evaluating the F1 score in correlation with the available positive samples during training, as shown in Fig. 5, two behaviors of AL were observed: For the instruments with high number of samples in EQS, vessel sealer and permanent cautery hook, AL was able to achieve higher F1-scores with fewer frames. For the remaining binary features AL was able to achieve better (azygos vein, metal clip applier, large clip applier & suction) or similar (gastric tube & scissors) F1-scores than EQS by selecting more samples of the feature. The correlations of the features blood and smoke are not evaluated in this way, because AL was not able to select frames of rare blood and smoke levels.

The results of the McNemar test comparing the correctness of the AL and EQS classifiers are shown in Table 2. The classifiers are compared for every feature and every cycle. No significant difference was found in any cycle for the features large clip applier, scissors, suction, or the anatomy features. The features vessel sealer, hook and metal clip applier showed significant differences mainly in the early and last cycles. Taking the performance metrics from Table 1 into account, a significant improvement of AL over EQS can be concluded for the instrument features, especially for the features vessel sealer and hook. The remaining blood and smoke levels differed significantly over almost all cycles. Cycle 6 showed an obvious cut as it was not significant in blood or any instrument feature. Although mostly not strongly reflected in the F1-scores, significant differences were found between the correctness of the classifiers for blood and smoke.

**Table 2:** McNemar test comparing the correctness of the classifiers using active learning (AL) or equidistant sampling (EQS) for frame selection [24]

Cycle	1	2	3	4	5	6	7	8	9	10
Blood	**0.010**	**0.045**	**< 0.001**	**< 0.001**	**< 0.001**	0.369	**< 0.001**	**< 0.001**	**< 0.001**	0.109
Smoke	**< 0.001**	**0.003**	0.770	**< 0.001**	**< 0.001**	**0.001**	**< 0.001**	0.516	**< 0.001**	**< 0.001**
Azygos vein	0.110	0.903	0.657	0.261	1.000	0.099	0.147	0.620	0.057	0.596
Gastric tube	0.488	0.770	1.000	0.677	0.532	0.775	0.643	0.560	1.000	0.868
Vessel sealer	0.253	**< 0.001**	0.061	0.123	0.404	0.134	0.230	**0.012**	**0.037**	**< 0.001**
Hook	0.810	0.787	**< 0.001**	0.871	0.307	0.453	0.081	0.201	0.110	**0.029**
Clip applier metal	**0.043**	1.000	**0.006**	**0.022**	0.628	0.149	0.789	0.077	**0.046**	0.077
Large clip applier		0.480				0.480	1.000	0.480	1.000	0.450
Scissors		0.480	0.248	0.683	1.000	1.000	0.683	1.000	0.617	0.617
Suction	0.109	0.296	0.584	1.000	0.860	0.169	0.814	1.000	0.332	0.663
Blood and smoke	**0.009**	0.669	**< 0.001**	**< 0.001**	0.226	**0.005**	**< 0.001**	**< 0.001**	**0.012**	**0.003**
Instruments	**0.021**	0.075	**< 0.001**	0.786	1.000	**0.008**	**0.030**	**0.004**	**< 0.001**	**< 0.001**
Anatomy	0.447	1.000	0.661	0.227	0.651	0.129	0.389	0.925	0.176	0.836
All features	0.234	0.262	**0.028**	**< 0.001**	0.387	**0.006**	**< 0.001**	**< 0.001**	**< 0.001**	**< 0.001**

The correctness of the AL and EQS classifications are compared over the 10 cycles using the McNemar test with Edwards correction. Significant differences (*p*-value < 0.05) are bold. Cycles with the exact same classification of both methods are left blank

An overall statistically significant difference in correctness of all classifiers (AL vs. EQS) could be shown with a superiority of AL taking the performance metrics from Table 1 into account.

### Surgomic report

**Fig. 6:**
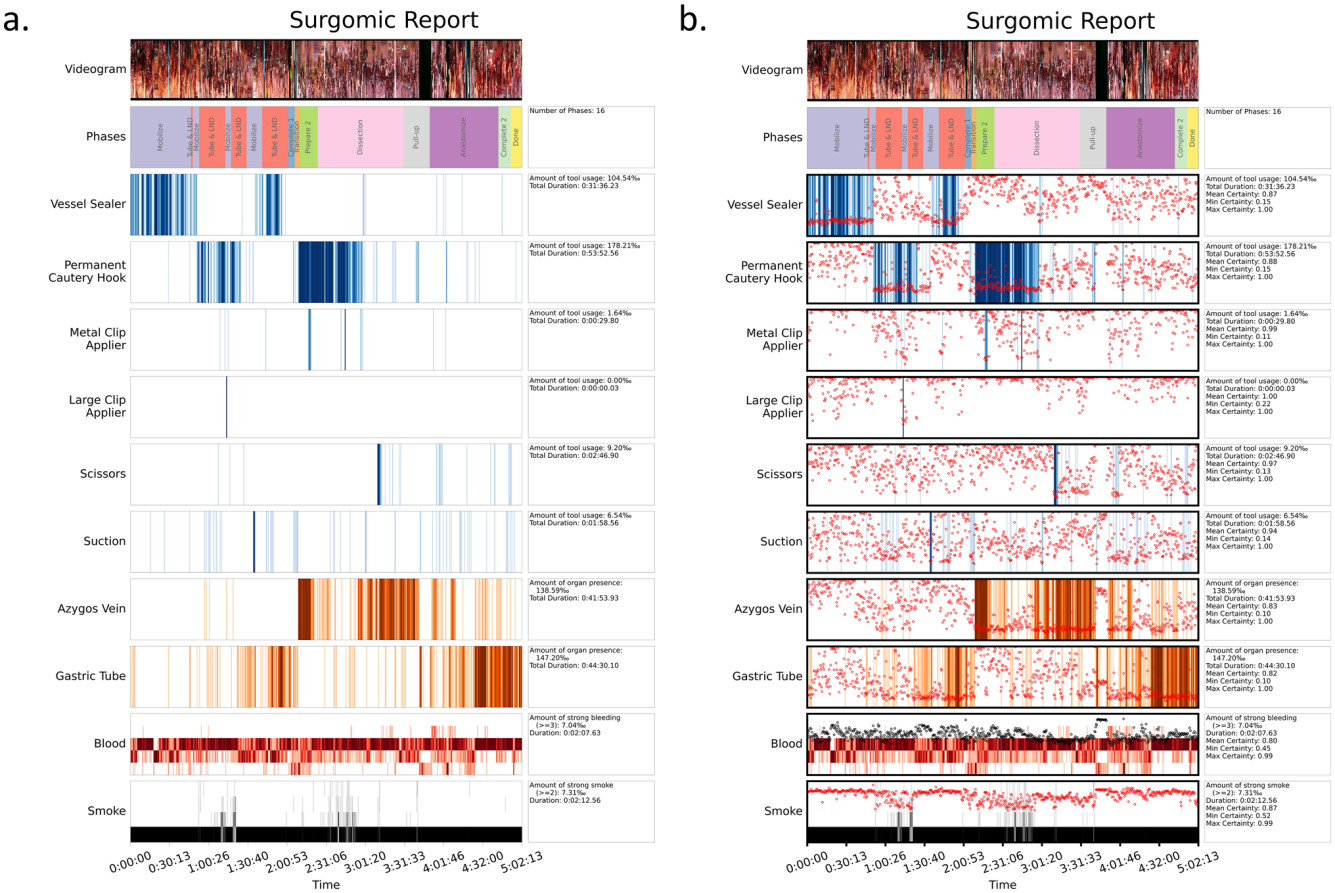
Surgomic report. The surgomic report is presented containing the automatically assessed feature information along the whole surgery (**a**). The report on the right (**b**) contains in addition the mean certainty of each prediction (red dots). A videogram is depicted on top. For the instrument/organ features the total duration of the features and the amount of tool usage/organ presence (in ‰) are shown. For the features “blood” and “smoke” the amount as well as the duration of a relevant amount of blood/smoke (levels > 2 for blood, > 1 for smoke) is shown (Color figure online)

The algorithms trained on all available annotated data were exemplarily applied on one video depicting the features in a surgomic report (Fig. 6).

### Live evaluation of surgomic features in the operating room

**Fig. 7:**
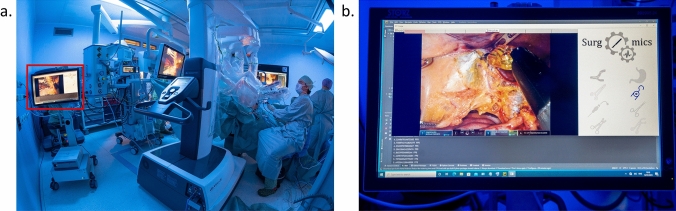
Live evaluation of Surgomics in the operating room. The surgomic feature tower was brought into the operating room (**a**) depicting the live detection of the surgomic features (**b**)

First test runs of surgomic feature predictions have been successfully performed live in the operating room at Heidelberg university hospital (Fig. 7). Neither during nor after the procedure were the results shown to the operating surgeon to not influence the treatment process. The preliminary recordings can be used for further model training and result replication. It was shown that the system provided a stable interface detecting features with 12 FPS. Basic live use in the operating room was demonstrated, further improvements for even more FPS and feature performance are possible and will be targeted in the future.

## Discussion

### Annotation and recognition of surgomic features

Based on the concept of Surgomics [10], in this study, we established an initial development process for surgomic features that explores AL as a method for frame selection and improvement of ML performance. Ten surgomic features from feature categories with high technical feasibility as well as clinical relevance according to Wagner et al. [10] were selected. Ordinal features (blood, smoke) were distinguished from binary features (anatomy and instruments). Automatic smoke evacuation using an industrial smoke-detection device has been reported by Takahashi et al. [27]. Furthermore, a binary classification of smoke/non-smoke images has been presented [28]. In our study, a four-level smoke classification was introduced which allows discrimination between the amount of smoke impairing the surgery or not. In addition, a five-level blood classification leaning on the work of Forrest et al. [14] was developed. As expected, the binary features were detected better than the more complex ordinal features. The comparatively low F1-scores of the ordinal features were also reflected in the greater disagreement among raters. Already during the design of the annotation protocol, feedback was received that a clear discrimination of the different levels was often difficult. Furthermore, subjectively it was also challenging to correctly annotate other features when there was a lot of smoke or blood in the image. As shown in our evaluation of inter-rater-agreement, for ordinal features three or even five raters were needed to reach acceptable inter-rater-agreement. Since low levels of blood and smoke are thought to have less surgical relevance, for further investigations the importance of inter-rater-agreement for low levels should be weighted lower. Instead, it should be considered not to annotate single frames regarding their level of blood and smoke, but sequences of clinically relevant bleeding that cause additional coagulation or sometimes even conversion to open surgery. Annotation rules of these sequences, which are highly reproducible across surgical centers, should increasingly be agreed upon. In this regard, annotation validation with board-certified surgeons should be addressed.

On the contrary, for binary features one-rater is likely to be sufficient for the annotation due to high inter-rater-agreement. To still realize annotation quality, two raters could be chosen in future studies and only in case of disagreement a third rater would be necessary.

### Active learning of surgomic features

Using AL in a prospective setting was able to prove an overall superiority over EQS and showed the most promising results for specific groups of features, especially the instrument group. AL chose specifically frames with rare instruments and needed less training frames while achieving higher F1-scores for the more frequent features vessel sealer and permanent cautery hook (Figs. 4 and 5). This data indicates that AL might achieve a certain saturation level when enough training frames are available enabling it to then better focus annotation efforts on other features.

Interestingly, although selecting more frames for the suction and the scissors compared to EQS (suction: 1065 frames with AL vs. 481 with EQS, scissors: 220 frames with AL vs. 68 with EQS), recognition results for EQS are slightly better than for AL. This counterintuitive result is probably explained by center-specific differences. For example, the suction is silver in Heidelberg videos, but black in Dresden videos. Also, AL selected way more frames from the Heidelberg dataset (1028 frames Heidelberg vs. 37 frames Dresden) in comparison to EQS (426 frames Heidelberg vs. 55 frames Dresden) what might explain the worse performance from AL on the more balanced test set (31 Heidelberg vs. 11 Dresden).

Overall, using AL it was possible to achieve very good results for some features with binary tag-annotation. However, the composition of the start data set proved to be an essential factor for AL. The start data set needs to contain a sufficient representation especially for rarely occurring features like high blood or smoke levels. This presented us with a great challenge. On the one hand, we wanted to try to reduce the annotation effort with AL as major objective of our study. The ability of selecting a diverse set of frames for desired labels would further replace AL of the ML algorithm by active selection via human experts that selects the difficult tasks. This would then increase the need for expert annotation resources that we aim to reduce and would thus counteract that very aim.

On the other hand, we needed sample frames for each feature and feature level for the start data set, while at the same time achieving a high diversity of frames. Further, the prospective setup of the experiment limited the amount of available videos during start set creation. We therefore decided to use an EQS method for creating the start data set and manually added missing feature levels so that at least every feature and feature level was represented. However, this proved to be insufficient for the AL algorithm in our experiment for high levels of blood and smoke. The number of frames in a start data set that AL requires to detect even more complex features, should be further investigated in future studies. The complexity of the features to be recognized as well as center-specific differences must be assessed before training starts and additional data for the start data set must be provided if necessary.

The minimum performance requirements to a model used for frames sampling via AL needs to be investigated to determine the minimum requirements to a start set.

Overall, in comparison to EQS, AL holds the potential to reduce the annotation effort for less complex features and to improve performance at the same time. Whether AL also provides an advantage for more complex features needs to be evaluated with further experiments where enough samples of rare classes can be found. Furthermore, the comparatively bad performance on blood and smoke indicates that new ML architectures should be investigated for ordinal-based features. The results achieved from the comparison study of AL vs. EQS for frame selection is also limited by the fact that the uncertainties of the models were not calibrated. The possible effects of calibrating the uncertainties needs to be investigated in future studies. In the context of the start set quality, the possibility of model bias needs to be mentioned. The used AL selection method is prone to bias as an existing bias is affecting the frame selection which then further enforces the bias. If a model is certain but wrong about the classification of a frame, the frame will not be selected, and the error not corrected. The possibility to tackle this issue with the use of modified selection methods should be investigated in future works.

### Next steps towards clinical application

A correlation of the surgomic features investigated in this study with clinical outcome has not yet been derived. Therefore, in the next step, validation in a prospective diagnostic trial should investigate correlation with clinically meaningful endpoints. Furthermore, the ordinal features should be further developed to improve recognition results of high levels. In this context it would be appropriate to take the ordinal nature of blood and smoke into account by applying methods specific for this data type, e.g., ordinal regression [29]. Since there is not enough data available from larger bleeding or smoking events, it should be considered to manually pick out and annotate certain video sequences. In this context, the potential role of AL should be further investigated. In the future, also the temporal context should be included into the evaluation to allow for a stronger focus on feature interactions. An example here could be that the permanent cautery hook causes heavy bleeding when cutting in the azygos vein during preparation. Consequently, the suction comes into the video and the hook is replaced by the clip applier. Consecutively, the bleeding can be controlled, the hook comes back into the video and surgery continues. It is these feature interactions and sequences that could be particularly relevant to predict postoperative complications [30]. For this purpose, the addition of the surgical phases and steps, in our case for RAMIE as further features is essential [31]. For example, a massive bleeding during the gastric mobilization phase could indicate an accidental injury of an abdominal artery whereas during the step of suturing the esophageal and gastric tube anastomosis bleeding is even desired, as this indicates good perfusion. Ideally, with the automatic detection of feature combinations, we will be able to derive a risk score or a specific recommendation for the surgeon. Especially regarding surgical skill assessment [32] and comparison between different centers, surgomic feature detection will be interesting. In this context, the features developed so far can serve as a basis for further investigations.

The surgomic report contains all features and can automatically be generated on a respective video, even directly after surgery. It presents the features in a comprehensible and clear manner and can quantitatively describe the procedure to make surgical progress measurable. If more features are developed in the future, the report will continuously be supplemented. A user evaluation with surgeons should be planned to assess potential benefits of the report in terms of surgical documentation and feedback.

## Conclusion

We presented ten different surgomic features automatically extracted from surgical video using machine-learning methods, even live in the operating room. Using the surgomic features, a surgomic report can automatically be generated summarizing information about the whole procedure. To speed up the development of new surgomic features, active learning can reduce annotation effort and improve algorithm performance compared to equidistant sampling for frame selection.

### Supplementary Information

Below is the link to the electronic supplementary material.

Supplementary file1 (DOCX 36149 kb)
