# Supplementary material for: Active learning for extracting surgomic features in robot-assisted minimally invasive esophagectomy: a prospective annotation study
Source: Surg Endosc. 2023 Oct 14;37(11):8577–93. doi: 10.1007/s00464-023-10447-6 (PMC10615926; doi:10.1007/s00464-023-10447-6)
Supplement: Supplementary file 1 — Supplementary file1 (DOCX 36149 kb) [file 464_2023_10447_MOESM1_ESM.docx]

# **Supplements**

**Supplement 1 - Annotation protocol for surgomic features**

| **Feature** | **Class/Level** | **Description** | **Examples** |
| --- | --- | --- | --- |
| Blood | 0 - No blood | Blood only in vessels. | 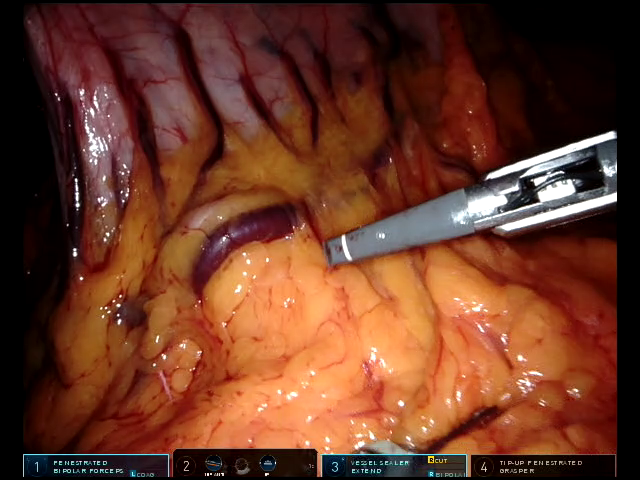 |
|  | 1 - Small amount of blood | Small hemorrhages on the surfaces visible and/or bloody gauze without a hemorrhage from the size of “blood accumulation” or bigger and/or already washed blood accumulation. Also, blood in the trocar is annotated as “small amount”.  (Suction, gauze, or swab are usually not needed, except suction used for liquid accumulation through washing) | 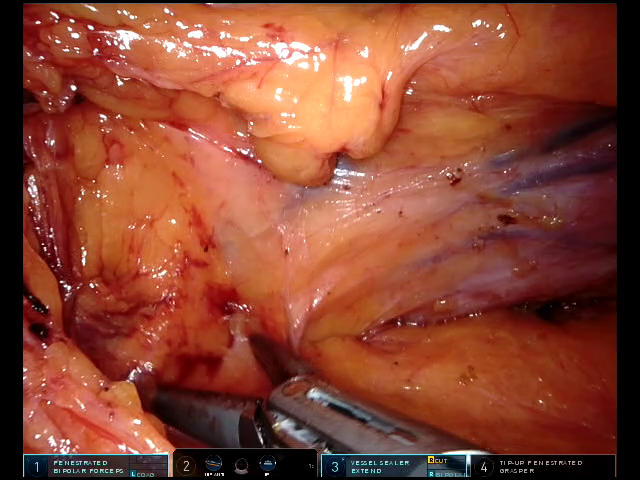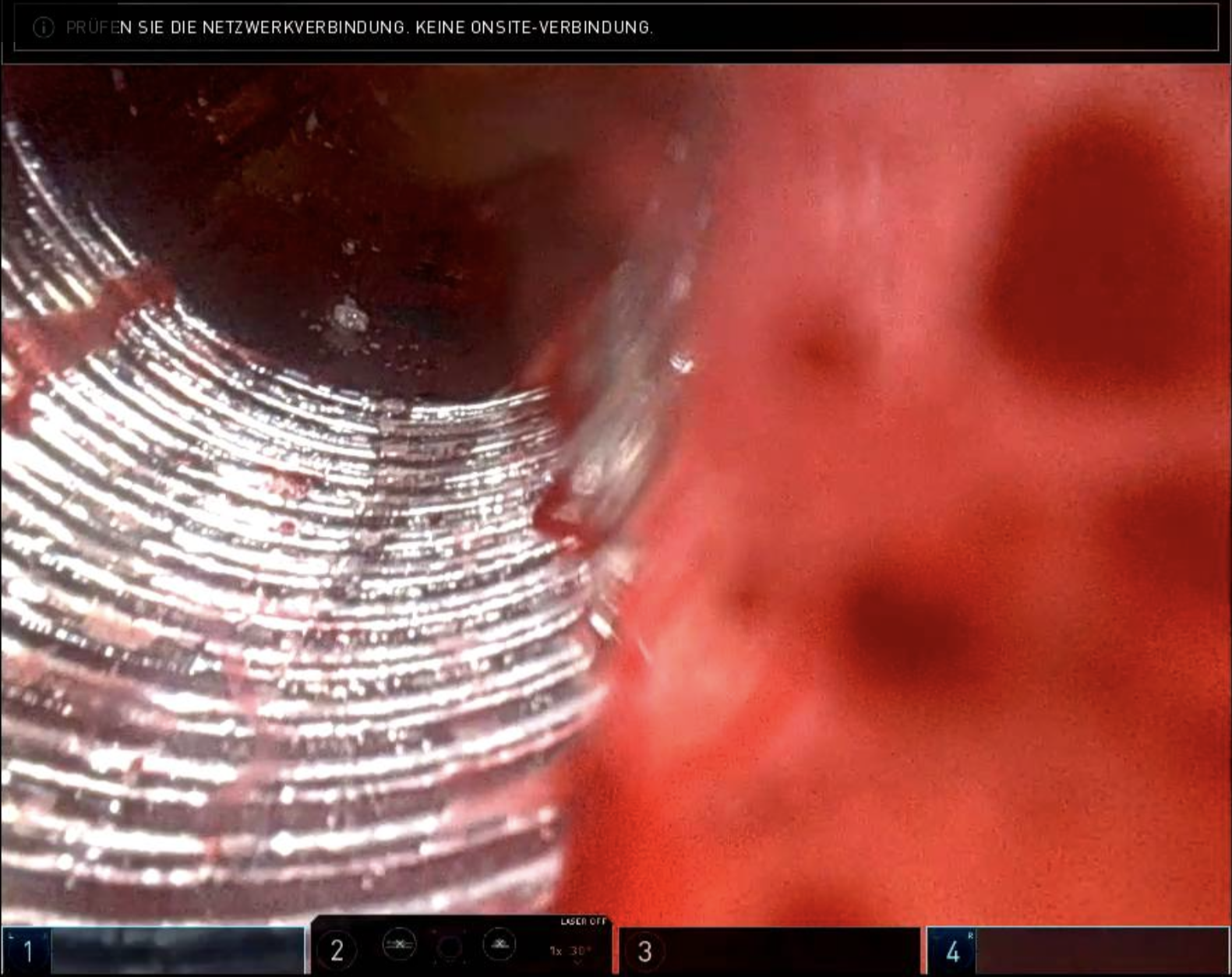 |
|  | 2 - Blood accumulation | Blood accumulation with a certain depth not just covering the surfaces.  (Suction, gauze, or swab can be used and are usually sufficient) | 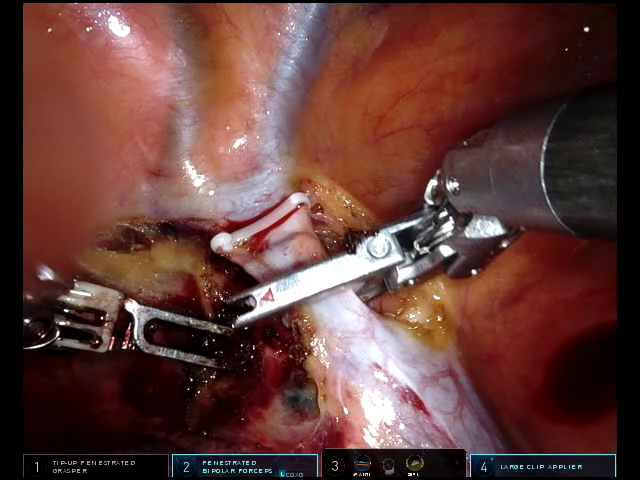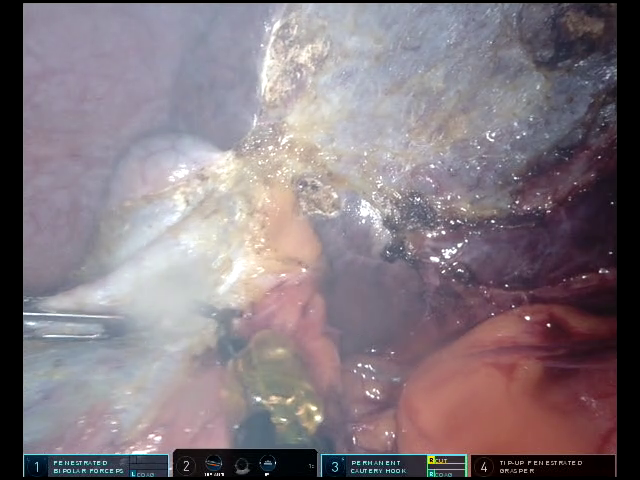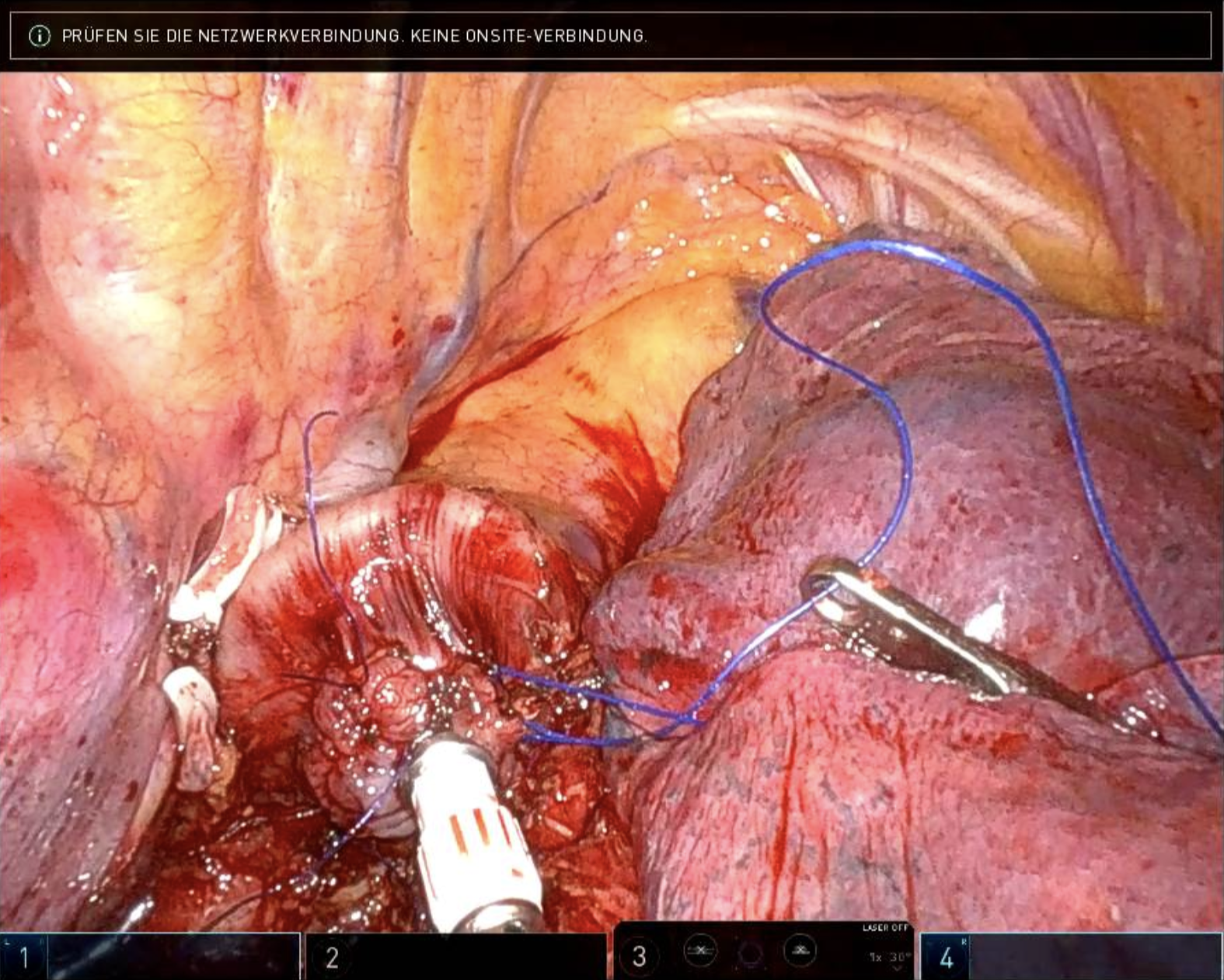 |
|  | 3 - Great amount of blood | Great puddle of blood and/or blood which may impair the surgical field.  (Suction during surgery is required, gauze and swab are usually not sufficient; source of bleeding might need to be identified and treated; potential danger to patient without immediate treatment) | 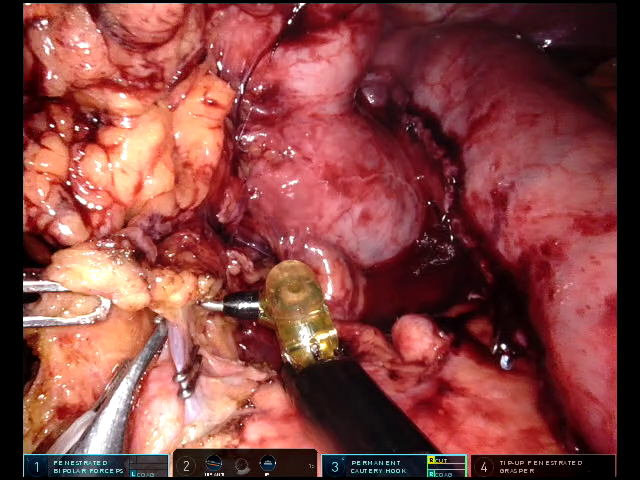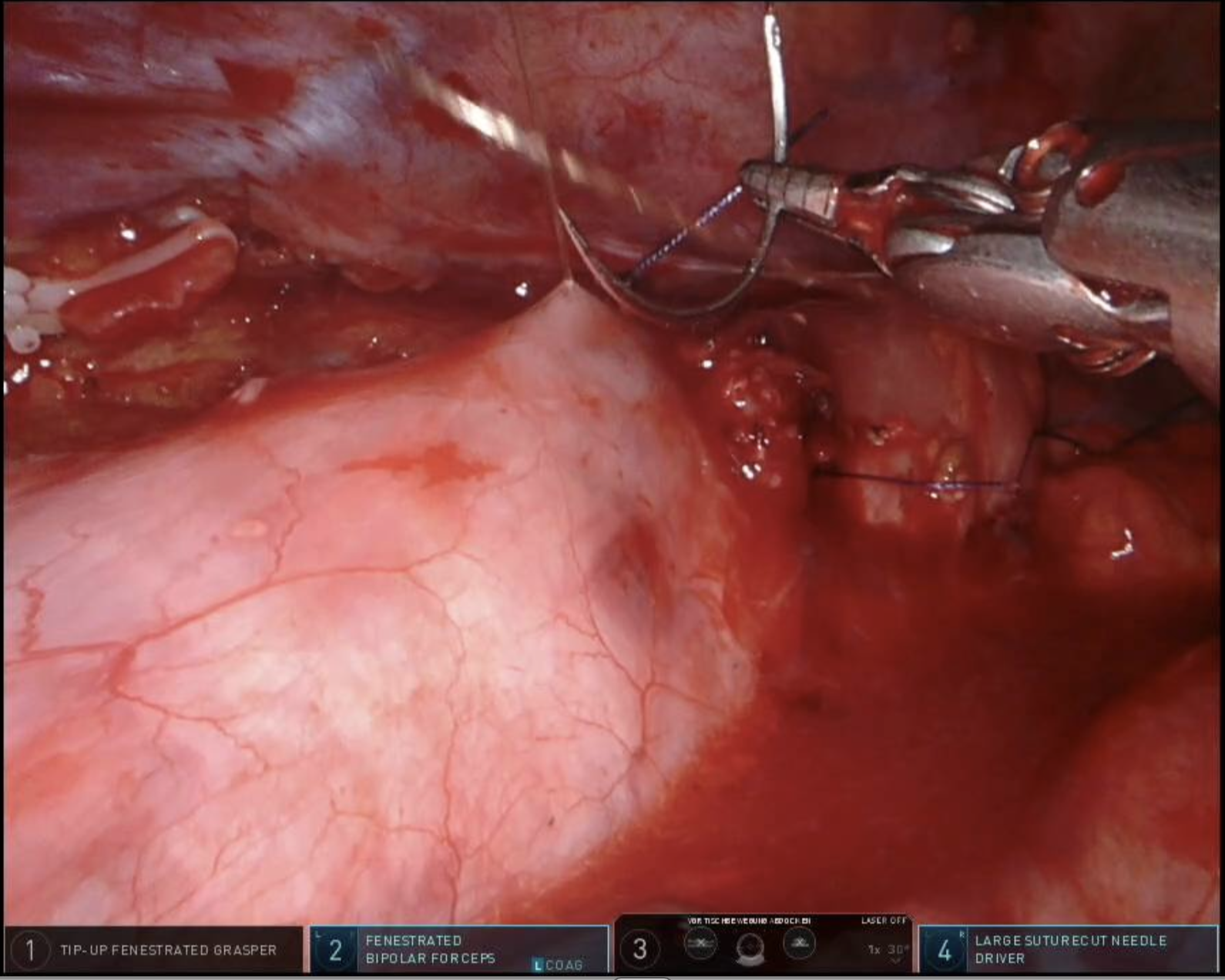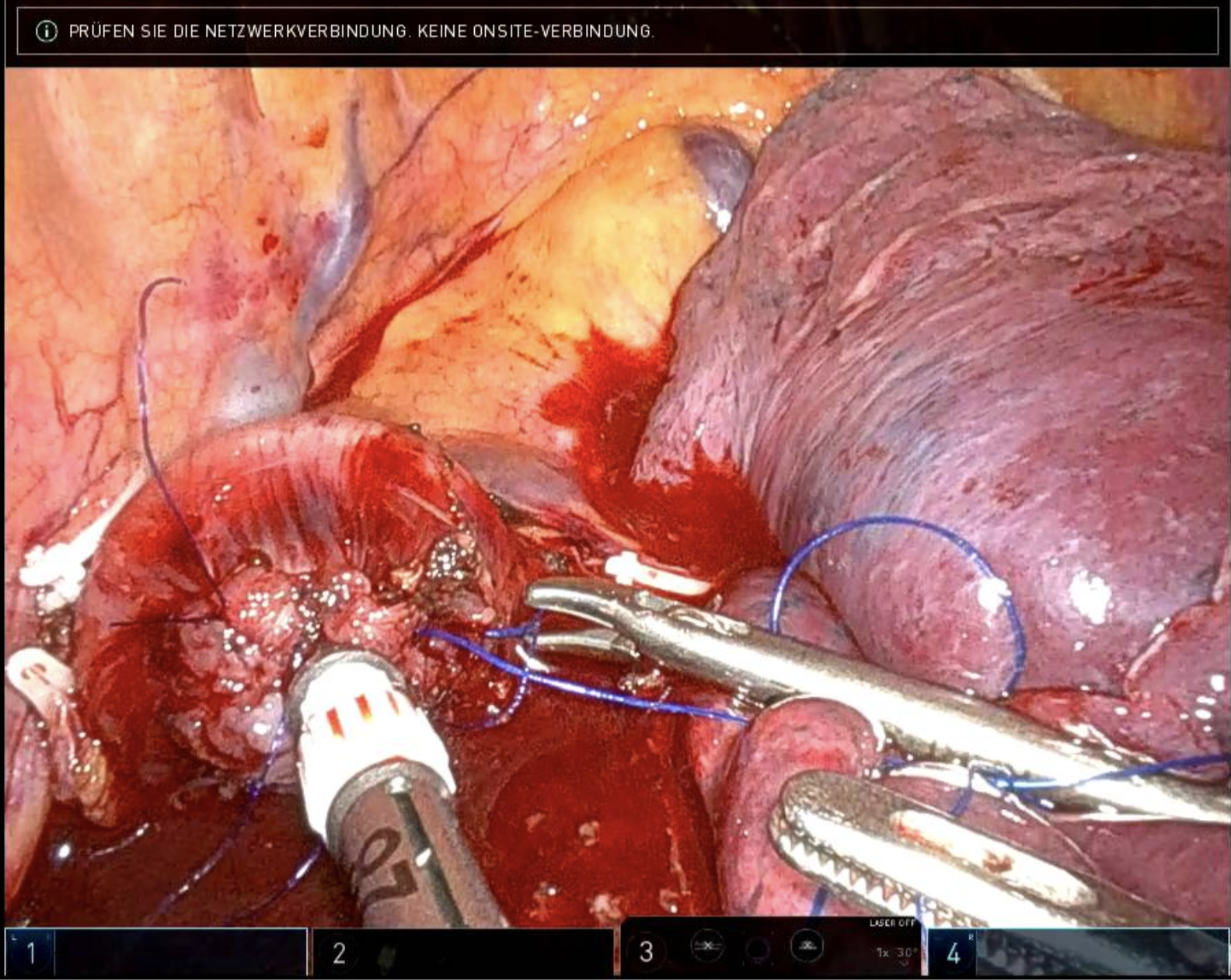 |
|  | 4 - Blood amount requiring immediate intervention | Spurting arterial hemorrhage where bleeding must be controlled using cauterization, clip or oversewing or major oozing bleeding where the source of bleeding must be identified and treated immediately with clip or oversewing (e.g., azygos vein).  (Potential danger to patient without immediate treatment) | 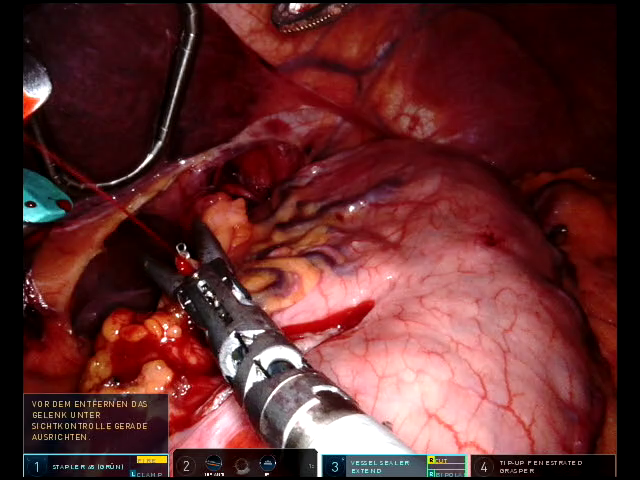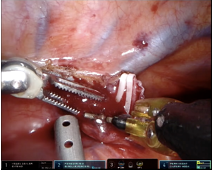 |
| Smoke | 0 - No smoke | Clear field of vision.  (Cauterizer usually not used) | 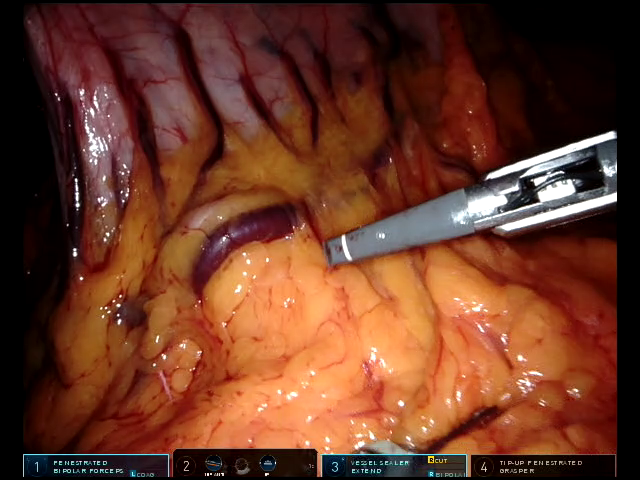 |
|  | 1 - Small amount of smoke | Field of view is slightly hazy, but does not impair the surgical working space and/or ascending smoke from the cauterizer in a certain area (local amount of smoke) | 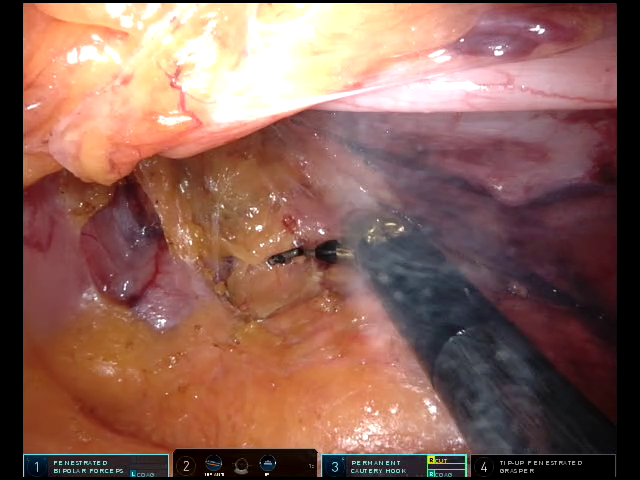 |
|  | 2 - Increased amount of smoke | The field of view is hazy and structures are not clearly distinguishable and the surgical working space is impaired (global amount of smoke), but still the procedure can be continued | 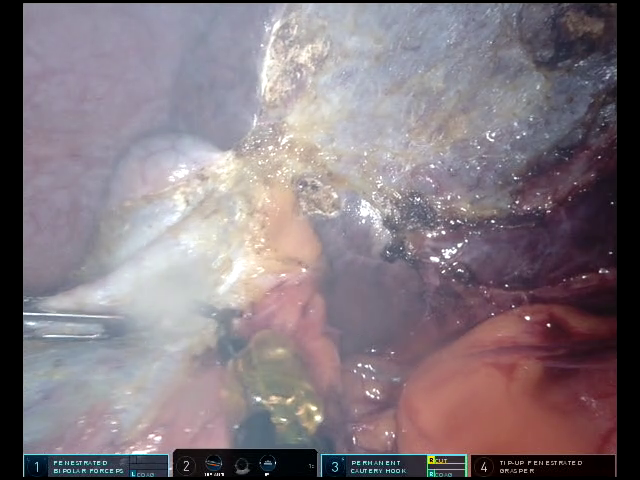 |
|  | 3 - Smoke amount leading to no visibility | The surgical field is not visible and/or the procedure needs to be stopped until the surgical field clears up again  (continuing the procedure may not be safe and might injure the patient)  (some instruments in the foreground may still be identifiable) | *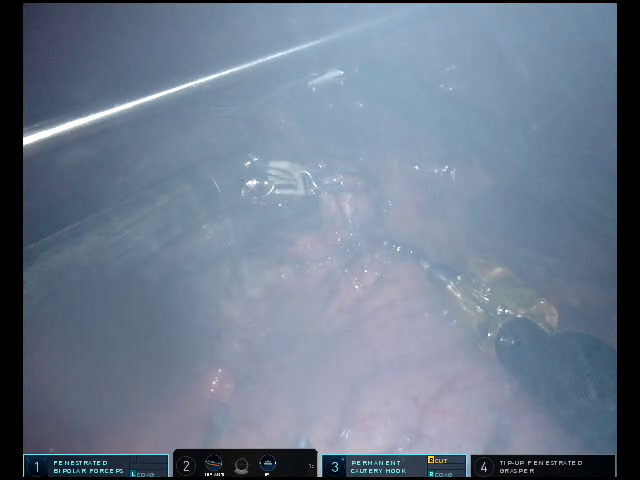* |
| Instruments | Permanent Cautery Hook | The Da Vinci Permanent Cautery Hook is visible in the frame and annotated with presence yes/no. | 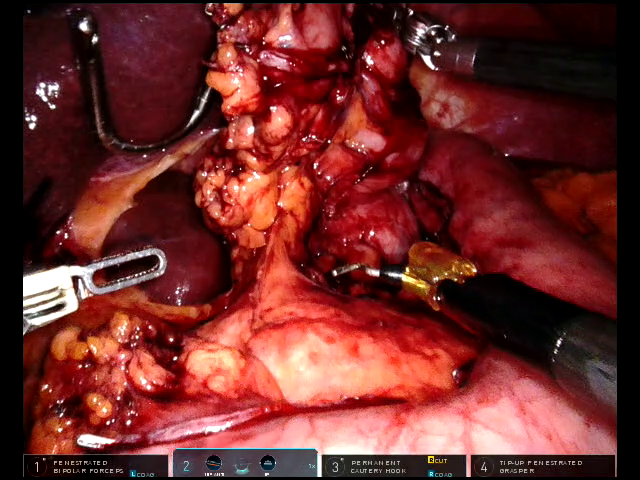 |
|  | Vessel Sealer Extend | The Da Vinci Vessel Sealer Extend is visible in the frame and annotated with presence yes/no. | 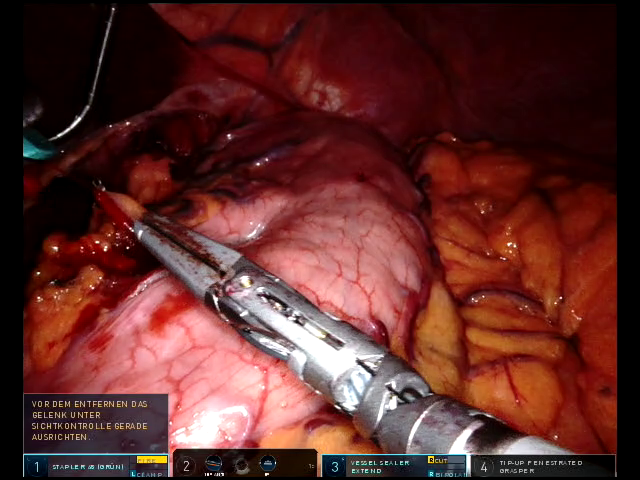 |
|  | Suction | The suction is visible in the frame and annotated with presence yes/no. | 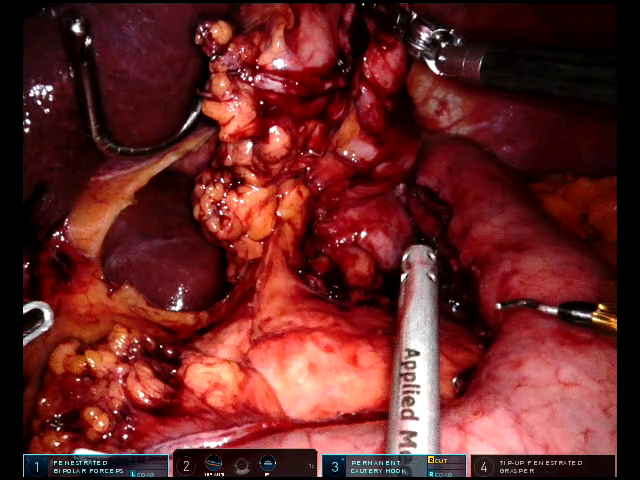 |
|  | Metal Clip Applier | The metal clip applier or the small metal clip applier is visible in the frame and annotated with presence yes/no. | 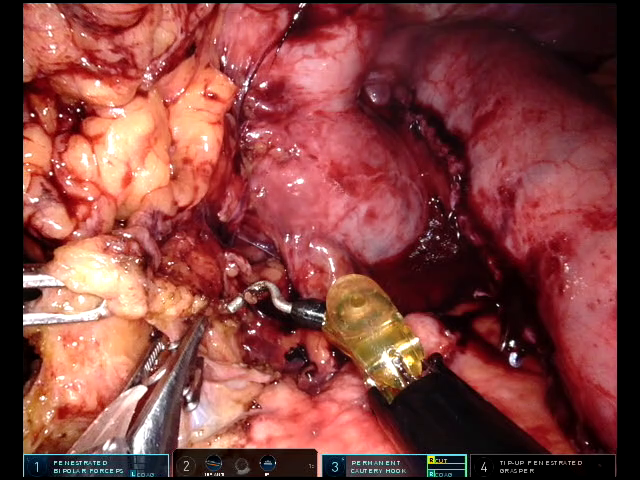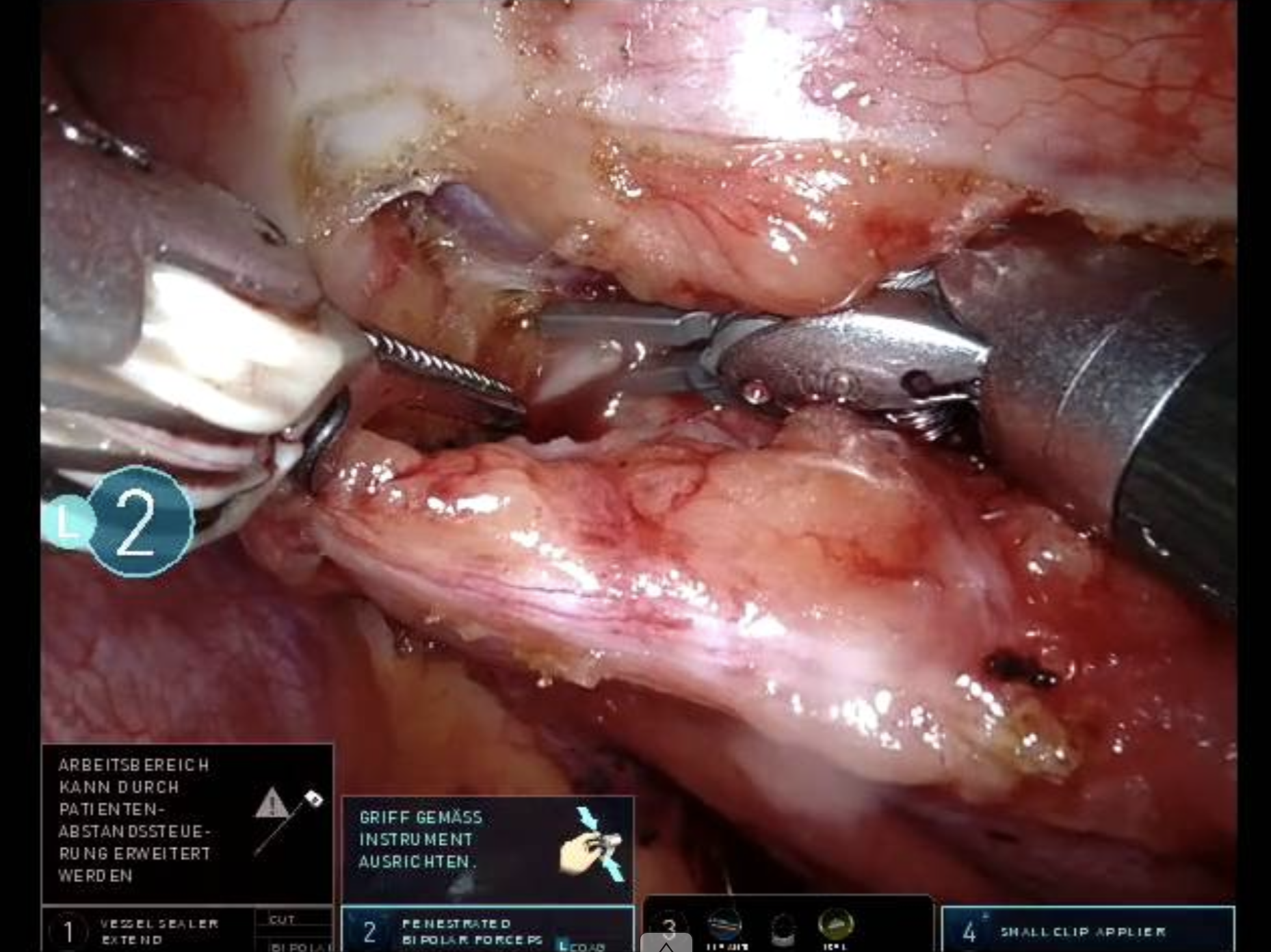 |
|  | Large Clip Applier | The large clip applier is visible in the frame and annotated with presence yes/no. | 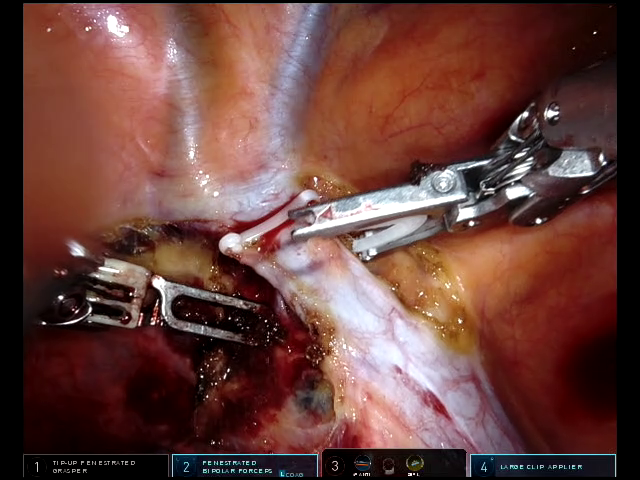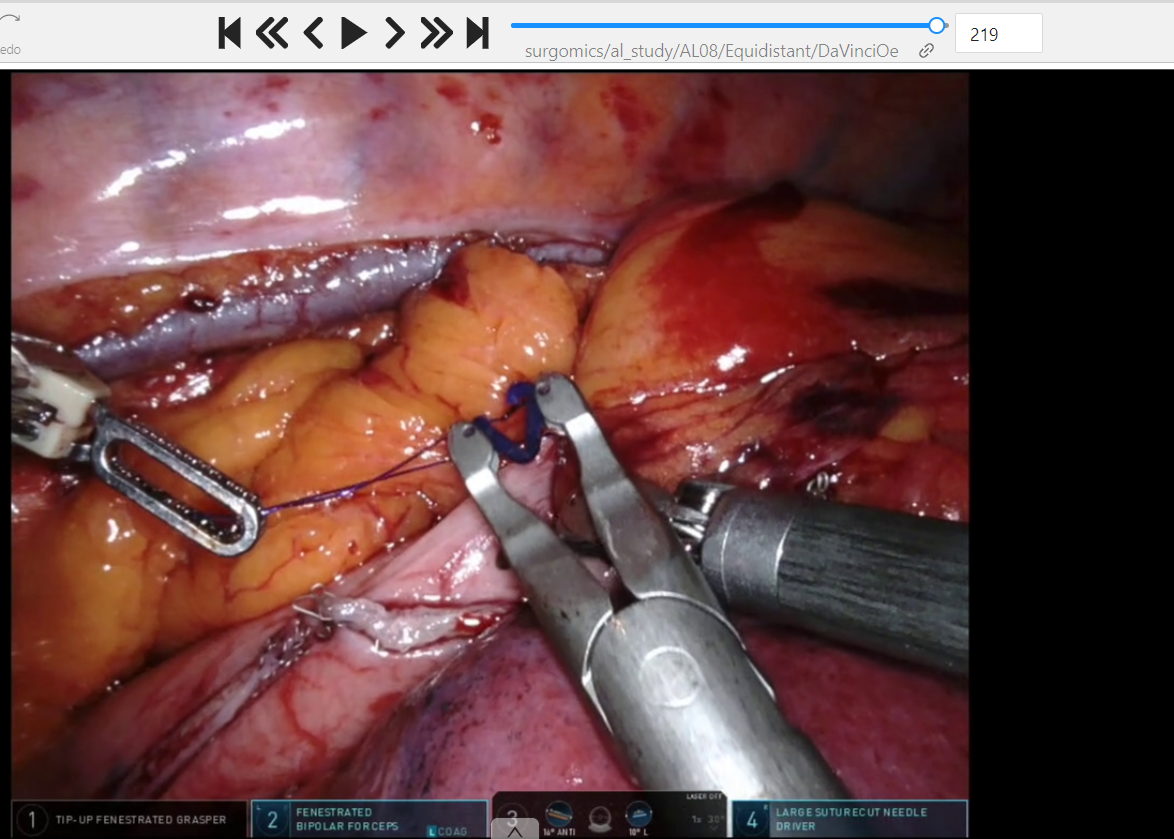 |
|  | Scissors | The Da Vinci Monopolar Curved Scissors or the laparoscopic scissors are visible in the frame. | 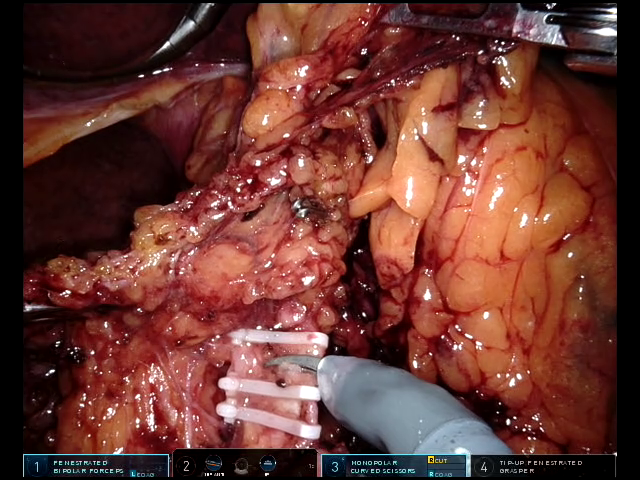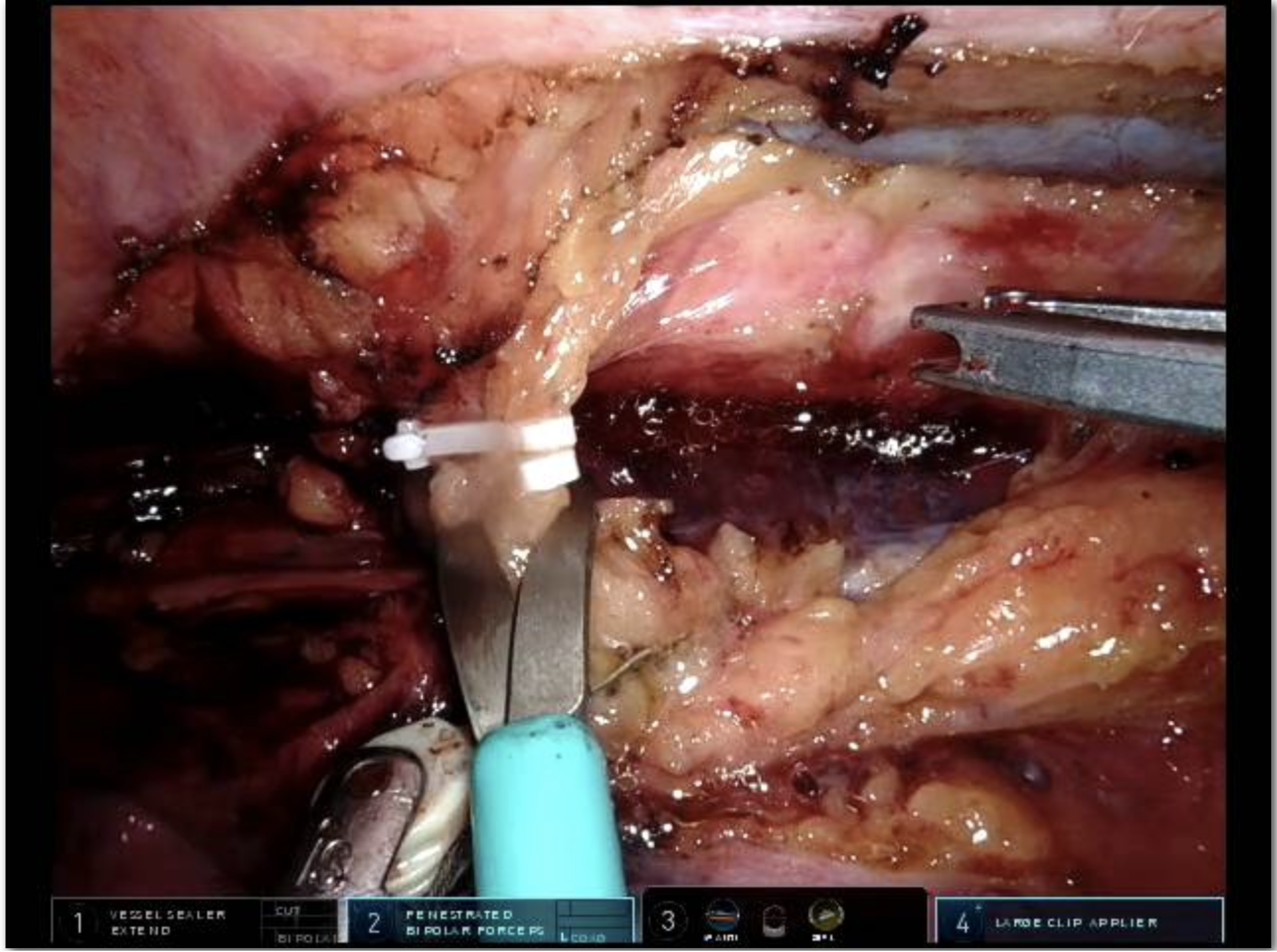 |
| Anatomical Structures | Gastric tube | The stapled gastric tube is visible in the frame and annotated with presence yes/no. The anatomic structure is defined as a gastric tube as soon as the linear stapler stapled for the first time in the stomach (first seam visible). The stapled stomach is considered as gastric tube as long as a stapled part of the stomach is present. Hence, when only the upper part of the stomach or the gastroesophageal junction is visible, the frame is annotated with presence “no”. | 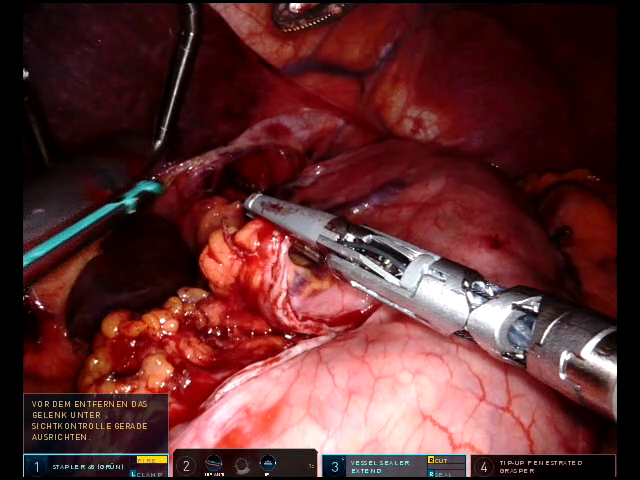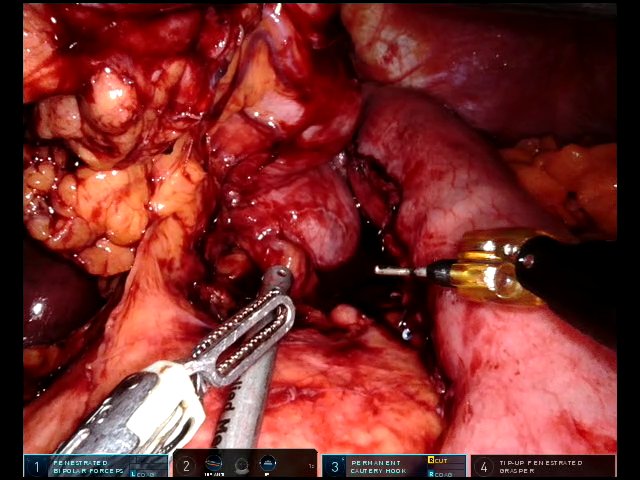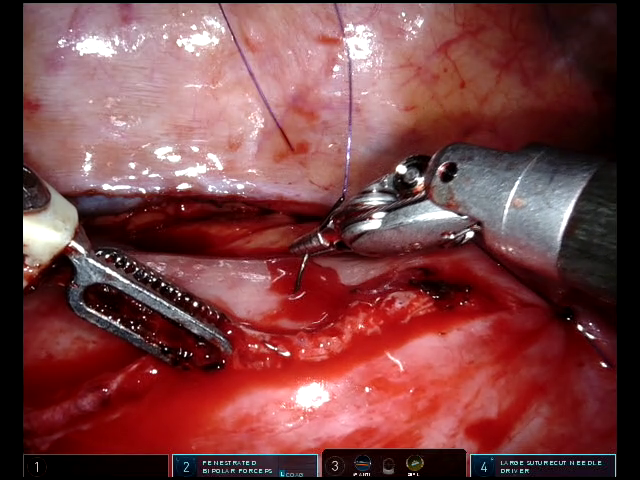 |
|  | Azygos Vein | The azygos vein and/or the azygos vein arcus is visible in the frame and annotated with presence yes/no. The clipped azygos vein arcus is annotated with presence “yes”. If the azygos vein is not yet dissected but its presence is only assumed under the covering tissue, the frame is annotated with presence “no”. | 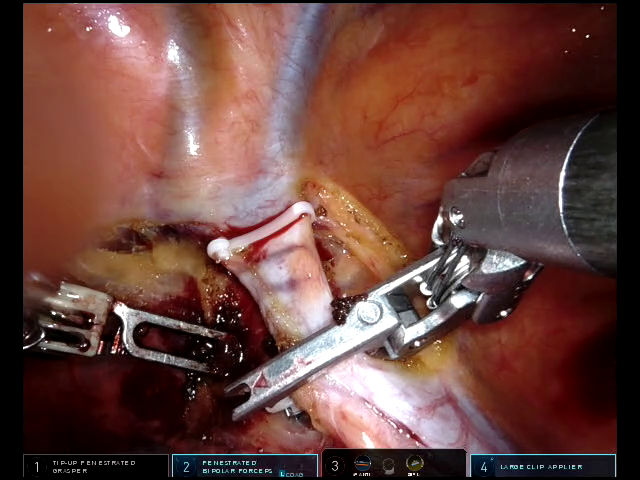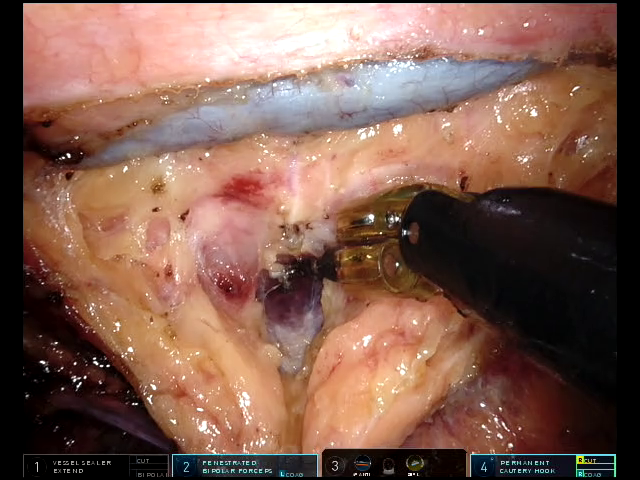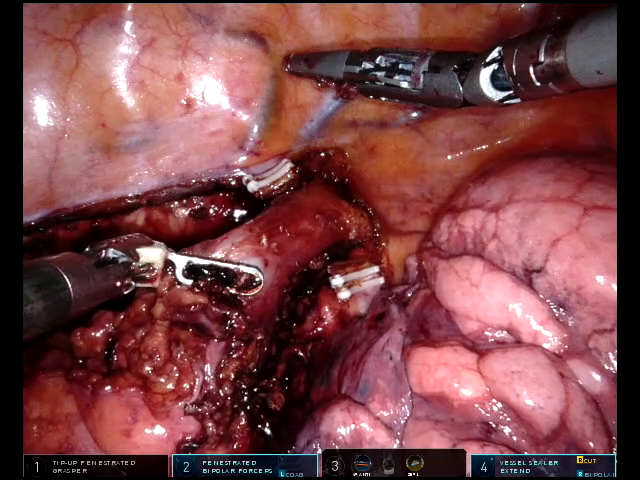 |
